# CH in the Solar Spectrum

**DOI:** 10.6028/jres.063A.002

**Published:** 1959-08-01

**Authors:** Charlotte E. Moore, Herbert P. Broida

## Abstract

CH lines in the solar spectrum have been identified by direct comparison of measured laboratory and solar wavelengths and intensities. The comparisons of individual rotational lines are included in a series of tables arranged according to electronic and vibrational transitions.

A detailed investigation of the presence of CH lines in the solar spectrum has been carried out recently as part of the preparation of a current edition of solar spectrum data to replace the 1928 compendium [1].^1^ Along with the study of atomic lines, a parallel survey of molecules in the sun is required for any complete revision of the “Solar Spectrum.” A preliminary résumé of the number of CH, OH, and CN lines in the solar spectrum was recently published [2].

Many lines of CH are familiar features in the solar spectrum. In his original table, Rowland [3] attributed some of these lines to “C,” and numerous CH lines were included in the 1928 edition [1]. Several authors have carried the work further. For example, Hunaerts [4] and Richardson [5] have each published a list of solar lines which may be ascribed to CH.

The present results are based on a study of the rotational structure of individual bands, together with relative laboratory intensities measured along the different branches. It reveals more CH lines in the sun than have been recognized heretofore. A summary of the counts is included in table 1. In this table the counts are handled differently than they were previously, in that the counts refer to individual laboratory lines; whereas in the earlier paper [2] they referred to solar lines. Consequently, if two CH lines contribute to one solar line, each CH line is counted here as a blend. Exception has been made in the case of table 10, where the laboratory lines are so closely blended that for some groups they are not counted as separate lines. The total number of laboratory lines given for this band is 119 ± to denote that the count is somewhat arbitrary. If only the leading lines of a band are present in the sun, the counts refer only to the stronger lines of the band, as for example in table 5, where only 31 lines out of 198 enter into the count.

The individual rotational bands are described in tables 2 to 11. Data on the laboratory analyses are given on the left hand side of each table, and the solar data are entered on the right. In table 6 only laboratory data are given, since this band is not observed in the sun.

Under the general heading, “Laboratory,” the respective rotational quantum numbers of the *P*, *Q*, and *R* branches are entered as indicated by the respective headings. Lines of weak satellite branches are indicated by the symbol ‡, and a summary of the satellite lines is included in footnotes to the tables.

These entries are followed by an intensity column. Intensities have been determined from measurements of the emission from the reaction zone of an acetylene-oxygen flame. A question mark (?) after the intensity number indicates a questionable intensity measurement caused by some overlapping from a strong neighboring line.(CH) indicates a weak line masked by a stronger CH line, whereas “M” indicates masking by some other emission in the laboratory source. In table 11 (OH) is entered as masking CH in some cases. Blank spaces in the intensity column result from lines too weak to be observed under the conditions used to obtain intensities.

Wavelengths have been derived from published wave numbers by means of Kayser’s Table [9]. In tables 2, 3, and 4 the laboratory analysis is from Gerö [6]; in tables 5 and 6 it is from N. H. Kiess and Broida [7]; in tables 7, 8, 9 from Gerö [6]; and in tables 10 and 11 from Heimer [8]. Fagerhölm’s measurements [10] of the A ^2^Δ—X^2^ Π and B ^2^Σ^−^—X ^2^Π bands do not fit the solar data so well as those of Gerö and have been used only as a check for errors. An asterisk in the wave number column is used to indicate a blend of two or more CH lines.

Under the general heading, “Sun,” the solar wavelengths are from the new solar ledger now in course of preparation. These wavelengths differ slightly from those published in 1928, because a running correction has since been made to reduce them to the 1928 International Solar Standards [11]. In addition, some new measurements have been averaged in for a number of lines. This work is in progress for the region short of 4000 A. Consequently, some solar wavelengths quoted in the present paper may differ slightly from those in the final solar ledger. The changes are so slight that they will not likely affect the CH identifications.

The second solar column contains the reduced equivalent widths of the solar lines furnished by Minnaert and his colleagues at the Utrecht Observatory. The equivalent widths have been measured in milliangstroms (mA) from the Utrecht Solar Atlas [12]. The tabular entries have been reduced from the measured values by dividing by X, and by allowing for the effect of blending when there is more than one contributor effective in producing the solar line. Details of the method are described by Minnaert in his 1951 paper [13]. These measured intensities replace the Rowland eye estimates for all solar lines except those in tables 10 and 11. In this spectral region the measured intensities have not yet been completed, and in their place the Rowland estimates are entered in brackets, —3 being substituted for Rowland’s 0000, *—*2 for 000, etc., as was done in 1928 [1].

In the solar column headed, “Spot,” the intensity behavior of the solar atomic lines in the spot spectrum is described as follows:
*d*double*S*greatly*N*diffusestrengthened*NN*very diffuse*u*unchanged*o*obliterated*w*weakened*s*strengthened*W*greatly weakened

These letters have been substituted for the estimated spot intensities published in more detail in 1933 [14]. Although the spot behavior was studied especially for atomic lines, in many cases it was noted in the 1933 paper when a line was a blend of an atomic and a molecular line in the spot spectrum as judged by the observed Zeeman effect, and the molecular contributor has turned out to be CH. An asterisk in this column indicates that the spot behavior refers to two adjacent solar lines; i.e., two disk lines are blended in the spot spectrum.

Following the spot column there is one headed “⊙–lab.” This is the difference, expressed in A, between the solar and laboratory wavelengths, another criterion used in making the identification of the solar line. Residuals greater than ±0.030 A are tolerated only if the solar line is very faint or blended, and in either case the CH identification is subject to question.

The next column gives the final adopted solar identification. Here the symbol |, preceding or following the chemical symbol, indicates that the line so marked is a stronger contributor to the solar line than other contributors. The symbol ‖ denotes a predominant contributor. Parentheses are used in this column for lines masked in the solar spectrum. Only the more important masked CH lines are thus noted in the tables. For those CH lines marked (CH) or (M) in the laboratory intensity column, if the solar identification refers to the CH line responsible for the masking, M is entered in the last column (see below).

In the last column four letters are used to indicate the judgment of the authors regarding the assigned identifications of the solar lines. They are as follows:

**P** The CH line is present in the solar spectrum, as indicated in the preceding column.
**B** The CH line is present in the solar spectrum but blended. A blend means that there are at least two contributors to the solar intensity.
**M** The CH line of the band in question is masked in the solar spectrum. This category includes CH lines too faint to contribute appreciably to solar lines. In some cases the solar identification may read “CH” but it refers to a CH line in another band.
**A** The CH line is absent from solar spectrum. Ends of branches at high *J*-values, where the line is too faint to be expected in the sun, are not counted as absent. Although there are a few wavelength agreements, these weak lines have been arbitrarily excluded from the count.

The present paper is one of a series planned to include the data on individual molecules present in the sun. A general survey of the molecular program was presented by the authors in Liège in 1956 [15]. It is hoped that the format adopted in the present tables may serve as a guide to those interested in preparing papers on molecular spectra in a style useful for astrophysical purposes.

The authors are most grateful to Mrs. Isabel D. Murray for her very accurate and painstaking work in helping to prepare the tables.

## Figures and Tables

**Table 1:** Summary

Table No.	Laboratory					Sun					
	Electronic transition	Vibrational transition	Wavelength range A	Total number lines	References	Strongest solar int. Δλ/λ	Summary of counts				
							Present	Blend	Masked	Absent	Total

2	A ^2^Δ–X ^2^Π	0, 0	4133 to 4413	309	6	39.6	155	92	46	16	309
3		1, 1	4185 to 4446	235	6	24.3	70	68	72	15	225
4		2, 2	4238 to 4468	188	6	26.6	54	36	61	8	159
5		0, 1	4726 to 4941	198	7	0.6	5	3	14	9	31
6		1, 2	4741 to 4913	149	7		0				0
				
			Total	1,079			284	199	193	48	724
7	B ^2^Σ^−^— X ^2^Π	0, 0	3871 to 4084	106	6	49.4	43	41	20	2	106
8		1, 0	3627 to 3710	54	6	13	18	10	22	4	54
9		1, 1	4025 to 4119	50	6	7.6	16	11	22	1	50
				
			Total	210		Rowl. est.	77	62	64	7	210
10	C ^2^Σ^+^—X ^2^Π	0, 0	3086 to 3219	119±	8	[2]	31	41	18	6	96
11		1, 1	3119 to 3222	50	8	[0]	8	13	24	5	50
				
			Total	169±			39	54	42	11	146

**Table 2:** CH in the Solar Spectrum A ^2^Δ— X ^2^Π (0,0)

Laboratory											Sun					

Q_1c_	Q_1d_	Q_2c_	Q_2d_	R_1dc_	R_1cd_	R_2dc_	R_2cd_	Intensity	Wave number cm^−1^	Wavelength A	Wavelength A	Disk int. Δλ/λ	Spot	⊙—lab. A	Solar identification	Notes
				35				1	24184.33	4133.743	4133.722	1.7		−0.021	CH? CN?	B
				34				2	24182.46	4134.063						A
						34		1.5	24181.09	4134.297	4134.347	20.3	*s*	+ 0.050	Fe i	M
				33				2	24175.90	4135.185	4135.173	1.5		−0.012	CH?	P
						33		2	24174.40	4135.441	4135.458	4.6	*uN*	+ 0.017	CH?—CN?	B
				32				2.5	24165.49	4136.966	4137.005	25.1	*u*	+ 0.039	Fe i	M
						32		2	24163.75	4137.264	4137.274	11.6	*s*	+ 0.010	Ti i	M
				31				3.5	24151.56	4139.352	4139.371	3.4		+ 0.019	CH	P
						31		3	24150.15	4139.594	4139.610	3.4		+ 0.016	CH	P
				30				4	24134.77	4142.232						A
						30		4	24133.31	4142.482	4142.474	11.6	*u*	−0.008	Cr i	M
				29				7	24115.36	4145.566	4145.559	7.7	*w*	−0.007	—CH	B
						29		6	24113.94	4145.810						A
				28				7.5	24093.65	4149.301	4149.370	26.8	*u*	+ 0.069	Fe i	M
						28		7	24092.32	4149.530	4149.538	2.9		+ 0.008	CH	P
					27			} 4	$\{\;\begin{array}{c} 24091.29\\ 24090.63 \end{array}$	4149.708	4149.699	1.0		−0.009	CH	P
							27			4149.821						A
				27				11	24069.93	4153.390	4153.389	8.7	*ud*?	−0.001	CH Fe i	B
						27		9.5	24068.63	4153.614	4153.620	5.8	s	+ 0.006	CH—	B
					26			} 12	$\{\;\begin{array}{c} 24064.65\\ 24064.24 \end{array}$	4154.301	4154.287	4.8	*u*	−0.014	CH	P
							26			4154.372	4154.379	7.0		+ 0.007	CH	P
				26				14	24044.50	4157.783	4157.788	30.5	*u*	+ 0.005	Fe i	M
						26		13	24043.24	4158.001	4158.007	7.0		+ 0.006	CH	P
					25			22	$\{\;\begin{array}{c} 24036.45\\ 24036.15 \end{array}$	4159.175	4159.186	26.7	*w*	+ 0.011	(CH)	M
							25			4159.227	4159.240	0.6		+ 0.013	CH	P
				25				17	24017.48	4162.460	4162.460	7.0	*o*?	0.000	|CH—CN?	B
						25		16	24016.32	4162.661	4162.660	8.2	*u*	−0.001	CH—CN?	B
					24			} 32	$\{\;\begin{array}{c} 24007.03\\ 24006.79 \end{array}$	4164.272	4164.263	8.9	*u*	−0.009	CN Fe i p—CH	B
							24			4164.314	4164.330	7.9	*u*	+ 0.016	CN CH	B
				24				21	23989.16	4167.374	4167.400	5.8		+ 0.026	CH	P
						24		20	23988.04	4167.569	4167.571	13.2	*u*	+ 0.002	CH	P
					23			} 44	$\{\;\begin{array}{c} 23976.39\\ 23976.15 \end{array}$	4169.594	} 4169.615	12.9	*ud*?	$\{\;\begin{array}{c} +0.021\\ -0.021 \end{array}$	} CH—CH	B
							23			4169.636						
				23				27	23959.62	4172.513	4172.482	12.0	*u*	−0.031	CH	P
						23		26	23958.65	4172.682	4172.644	25.4	u*	−0.038	Fe i	M
					22		22	58	23944.63*	4175.125	4175.130	15.8	*wN*	+ 0.005	CH—CN	B
				22				36	23929.08	4177.838	4177.849	11.5	*wN*	+ 0.011	|CH—CN	B
						22		33	23928.19	4177.994	4177.999	2.4		+ 0.005	CH	P
					21		21	73	23912.18*	4180.791	4180.811	19.1		+ 0.020	CH	P
				21				42	23897.65	4183.333	4183.326	8.6	*u*	−0.007	Ti i—CH	B
						21		40	23896.84	4183.475	4183.457	18.6	*u*	−0.018	|V ii—CH	B
					20		20	92	23878.86*	4186.625	4186.622	22.7	*uN*	−0.003	Ce ii—CH i	B
				20				58	23865.52	4188.965	4188.978	17.2		+ 0.013	Ni i—CH	B
						20		50	23864.77	4189.096	4189.102	11.0		+ 0.006	CH	P
					19		19	108	23845.01*	4192.568	4192.572	15.7	*u*	+ 0.004	CH	P
				19				70	23832.66	4194.741	4194.736	10.5	*uN*	−0.005	CN—CH	B
						19		67	23831.96*	4194.864	4194.848	15.5	*u*	−0.016	CH	P
					18		18	128	23810.55*	4198.636	4198.638	30.5	*u*	+ 0.002	CH—Fe i	B
				18				90	23799.25	4200.629	4200.601	13.3		−0.028	CH	P
						18		90	23798.74	4200.719	4200.700	7.1	*u?*	−0.019	CH	P
					17		17	136	23775.85*	4204.764	4204.753	16.4		−0.011	CH	P
				17				} 114	$\{\;\begin{array}{c} 23765.48\\ 23765.15 \end{array}$	4206.599	4206.578	10.5	*u*	−0.021	CH	P
						17				4206.657	4206.702	30.0	*s*	+ 0.045	|Fe i CH	B
					16		16	142	23740.66*	4210.997	4210.967	23.7		−0.030	CH	P
				16				} 146	$\{\;\begin{array}{c} 23731.43\\ 23731.18 \end{array}$	4212.634	} 4212.642	22.1	*uN*	$\{\;\begin{array}{c} +0.008\\ -0.037 \end{array}$	} CH–CH	B
						16				4212.679						
							15	} 142	$\{\;\begin{array}{c} 23705.74\\ 23705.45 \end{array}$	4217.200	4217.214	14.0		+ 0.014	CH	P
					15					4217.251	4217.268	14.0		+ 0.017	CH	P
				15		15		188	23697.18*	4218.723	4218.725	18.5		+ 0.002	CH	P
							14	} 136	$\{\;\begin{array}{c} 23670.48\\ 23669.97 \end{array}$	4223.482	4223.486	13.5		+ 0.004	CH	P
					14					4223.573	4223.574	15.9		+ 0.001	CH	P
				14		14		192	23662.79*	4224.854	4224.860	22.7		+ 0.006	CH Cr ii	B
							13	124	23635.22	4229.782	4229.774	27.2	*s*	−0.008	Fe i (CH)	M
					13			120	23634.53	4229.906	4229.914	16.3		+ 0.008	CH	P
				13		13		197	23628.33*	4231.016	4231.026	22.4	*u*	+ 0.010	Ni i (CH)	M
							12	128	23599.94	4236.106	4236.122	12.0		+ 0.016	CH	P
					12			130	23599.14	4236.249	4236.263	23.8		+ 0.014	CH	P
						12		} 182	$\{\;\begin{array}{c} 23594.07\\ 23593.74 \end{array}$	4237.160	4237.182	28.8	*u*	+ 0.022	Fe i CH	B
				12						4237.219	4237.254	14.2	*s?*	+ 0.035	CH	P
							11	142	23564.69*	4242.443	4242.455	20.0		+ 0.012	CH	P
					11			132	23563.71	4242.619	4242.604	20.5	*u*	−0.015	Fe i—CH	B
						11		154	23559.70	4243.341	4243.365	35.6	*u*	+ 0.024	CH—Fe i	B
				11				156	23559.04	4243.460	4243.454	16.3	*u?*	−0.006	CH	P
							10	132	23529.79	4248.736	4248.726	19.1		−0.010	CH	P
					10			140	23528.63	4248.945	4248.944	16.5		−0.001	CH	P
						10		144	23525.58	4249.497	4249.494	14.6		−0.003	CH	P
				10				160	23524.74*	4249.648	4249.637	23.0	*ud*	−0.011	—CH	B
							9	136	23495.17*	4254.996	4254.979	27.0	*ud*	−0.017	Fe i—CH	B
					9			144	23493.76	4255.252	4255.251	16.9		−0.001	CH	P
						9		136	23491.66	4255.632	4255.637	16.0		+ 0.005	CH	P
				9				140	23490.58	4255.828	4255.839	19.5	*s*	+ 0.011	CH—Fe i	B
							8	136	23460.81	4261.228	4261.223	16.0		−0.005	CH	P
					8			156	23459.13	4261.533	4261.531	16.4	*w*	−0.002	CH	P
						8		140	23458.02	4261.734	4261.738	15.7	*wd?*	+ 0.004	CH	P
				8				154	23456.59	4261.995	4261.978	14.5		−0.017	CH	P
	31							9	23437.17	4265.526	4265.542	4.7	*w*	+ 0.016	CH—	B
			31					8	23436.50	4265.648	4265.679	5.9	*s*	+ 0.031	Ti i	M
	30							16?	23431.23	4266.607	4266.623	4.7	*w?*	+ 0.016	CH	P
			30					30?	23430.54*	4266.733	4266.742	10.3		+ 0.009	CH	P
							7	134	23426.93	4267.391	4267.389	14.1		−0.002	CH	P
					7			} 270	$\{\;\begin{array}{c} \;23424.91*\\ 23424.68 \end{array}$	4267.759	4267.749	13.1	*uN*	−0.010	CH	P
						7				4267.801	4267.827	26.7	*u*	+ 0.026	|Fe i CH	B
			29	7				168	23422.97*	4268.112	4268.112	17.8		0.000	CH	P
	28							M	23415.01	4269.563	4269.585	7.2	*u*	+ 0.022	CH	P
			28					M	23414.29	4269.694	4269.740	17.8	*u*	+ 0.046		M
	27							7?	23405.14	4271.363	4271.374	16.2	*u*	+ 0.011	CH	P
			27					6?	23404.63	4271.456	4271.460	10.3		+ 0.004	CH	P
	26							12?	23394.50	4273.306	4273.332	21.5	*u*	+ 0.026	|Fe ii Ti i (CH)	M
			26				6	138	23393.53*	4273.483	4273.485	14.3		+ 0.002	CH	P
						6		152	23391.87	4273.786	4273.797	14.0		+ 0.011	CH	P
					6			166	23391.10	4273.927	4273.942	14.0	ud*	+ 0.015	CH	P
				6				152	23389.65	4274.192	4274.193	15.2		+ 0.001	CH	P
27								(CH)	23383.88*	4275.247	4275.258	15.0		+ 0.011	CH	M
	25							(CH)	23383.17*	4275.377	4275.386	24.3		+ 0.009	CH	M
		27	25					M	23382.70*	4275.463						A
26								28	23374.27	4277.005	4276.995	9.8	*sd*	−0.010	V i—CH	B
		26						25	23373.07	4277.224	4277.233	8.6		+ 0.009	CH—	B
	24							} 71	$\{\;\begin{array}{c} 23371.49\\ 23371.24 \end{array}$	$\{\;\begin{array}{c} 4277.513\\ 4277.559 \end{array}$	4277.535	16.8	*ud*	$\{\;\begin{array}{c} +0.022\\ -0.024 \end{array}$	CH—CH	B
			24													
25								36	23364.18	4278.852	4278.853	10.8	*u*	+ 0.001	|CH—Ce ii?	B
		25						35	23363.01	4279.066	4279.067	9.1	*ud*	+ 0.001	CH Mo ii	B
							5	130	23360.76	4279.478	4279.490	14.7	*u*	+ 0.012	Fe i CH	B
	23		23			5		220	23359.51*	4279.707	4279.720	21.7		+ 0.013	CH	P
					5			174	23357.76*	4280.028	4280.037	15.0	*wd?*	+ 0.009	CH	P
				5				159	23356.73	4280.217	4280.220	16.4	*w*	+ 0.003	CH Fe i	B
24								56	23353.65*	4280.781	4280.788	13.7		+ 0.007	Sm ii CH	B
		24						60?	23352.70*	4280.956	4280.964	11.4		+ 0.008	CH	P
	22		22					112	23347.15*	4281.974	4281.972	17.7		−0.002	CH	P
23								60	23342.69	4282.792	4282.796	13.1	*w*	+ 0.004	CH	P
		23						56	23341.68	4282.977	4283.014	31.0	*s*	+ 0.037	Ca i	M
	21		21					142	23334.86*	4284.228	4284.228	20.1	*w*	0.000	|CHCr ii	B
22								70	23331.56	4284.835	4284.837	11.2		+ 0.002	CH	P
		22						80	23330.64	4285.003	4285.008	17.5	*s*	+ 0.005	CH Ti i|	B
							4	150	23328.63*	4285.373	4285.371	7.9		−0.002	CH	P
						4		120	23327.79	4285.527	4285.538	15.4		+ 0.011	CH	P
					4			180	23324.82	4286.073	4286.090	6.8		+ 0.017	CH	P
				4				156	23324.15	4286.196	4286.196	14.5		0.000	CH	P
	20		20					238	43322.56*	2286.488	4286.477	26.6	*uN*	−0.011	Fe i—CH	B
21								80?	23320.37	4286.891	4286.884	13.8	*u*	−0.007	Fe i (CH)	M
		21						80?	22319.57	4287.038	4287.051	6.5	*u?*	+ 0.013	CH	P
	19		19					204	23310.40*	4288.724	4288.736	17.5		+ 0.012	CH	P
20								110	23309.13	4288.958	4288.962	17.2	*u*	+ 0.004	|Fe i CH	B
		20						94	23308.46	4289.081	4289.080	17.0	*s*	−0.001	Ti i (CH)	M
	18		18					310	23298.36*	4290.941	4290.956	31.9	*s*	+ 0.015	Ti i (CH)	M
19								} 300	$\{\;\begin{array}{c} 23298.00\;\\ 23297.42*\\ 23296.85* \end{array}$	4291.007	4291.019	10.2		+ 0.012	CH	P
		19					3			4291.113	4291.121	26.1	*u*	+ 0.008	CH	P
						3				4291.219	4291.220	14.2	*u*	+ 0.001	CH Ti i	B
								8	23293.17*	4291.897						A
					3			} 260	$\{\;\begin{array}{c} \;23292.36*\\ 23291.96 \end{array}$	4292.046	4292.055	12.3		+ 0.009	CH	P
				3						4292.119	4292.129	22.1	*u?*	+ 0.010	CH—Fe i	B
18								} 400	$\{\;\begin{array}{c} 23287.07\;\\ 23286.63* \end{array}$	4293.021	4293.036	15.1		+ 0.015	CH	P
	17	18	17							4293.102	4293.114	20.5		+ 0.012	CH	P
17		17						} 240	$\{\;\begin{array}{c} 23276.29\\ 23276.04 \end{array}$	4295.009 4295.055	} 4295.040	23.0		$\{\;\begin{array}{c} +0.031\\ -0.015 \end{array}$	} CH—CH	B
			16					} 260	$\{\;\begin{array}{c} 23275.33\\ 23275.07 \end{array}$	4295.186	} 4295.226	24.9		$\{\;\begin{array}{c} +0.040\\ -0.008 \end{array}$	} CH—CH	B
	16									4295.234						
							2	} 200	$\{\;\begin{array}{c} 23267.58\\ 23267.37 \end{array}$	4296.617	4296.584	24.4	*w*	−0.033	Fe ii (CH)	M
						2				4296.656	4296.683	13.3	*sN*	+ 0.027	CH—	B
16		16						280	23265.75*	4296.955	4296.956	25.6		+ 0.001	CH	P
			15					} 250	$\{\;\begin{array}{c} 23264.34\;\\ 23263.96* \end{array}$	4297.215	4297.219	22.3		+ 0.004	CH	P
	15									4297.286	4297.291	20.9		+ 0.005	CH	P
							‡	8	23261.34*	4297.769	4297.751	12.8	*sN*	−0.018	‖‖ ‖Cr i V i?	M
				2	2			240	23260.14*	4297.991	4297.979	7.4	*s*	−0.012	CH	P
15		15						340	23255.76*	4298.801	4298.813	22.0	*u*	+ 0.012	CH	P
			14					220	23253.88*	4299.148	4299.138	24.2	*u?*	−0.010	CH	P
	14							214	23253.34*	4299.248	4299.249	49.3	*sN*	+ 0.001	|Fe i Ti i (CH)	M
						1	1	160	23247.54*	4300.321	4300.318	20.2	*w*	−0.003	CH	P
14		14						370	23246.12*	4300.583	4300.573	29.3	*S*	−0.010	‖ Ti i—CH	B
			13					200	23243.86	4301.001	4301.000	23.7	*uN*	−0.001	CH	P
	13							220	23243.12*	4301.138	4301.174	18.1	*uN?*	+ 0.036	CH—Cr i|	B
		13						} 324	$\{\;\begin{array}{c} 23237.11\\ 23236.84 \end{array}$	4302.251	} 4302.297	31.4		$\{\;\begin{array}{c} +0.046\\ -0.004 \end{array}$	} CH—CH	B
13										4302.301						
			12					300	23234.38*	4302.756	4302.754	24.2		−0.002	CH	P
	12							200	23233.54	4302.912	4302.913	19.0		+ 0.001	CH	P
							‡	M	23229.71*	4303.621	4303.595	15.1	*s*	−0.026	Nd ii	M
		12						} 450?	$\{\;\begin{array}{c} 23228.59*\\ 23227.95* \end{array}$	4303.829	4303.835	28.6	*u*	+ 0.006	CH	P
12				1	1					4303.947	4303.937	27.6	*wN*	−0.010	—CH	B
			11					190	23225.55	4304.392	4304.395	16.0		+ 0.003	CH	P
	11							216	23224.53*	4304.581	4304.571	25.1	*w*	−0.010	Fe i—CH	B
		11						208	23220.57	4305.315	4305.322	13.5		+ 0.007	CH	P
11								204	23219.85	4305.449	4305.456	28.8	*u*	+ 0.007	|Fe i Sr ii (CH)	M
			10					300	23217.39	4305.905	4305.918	36.2	*S*	+ 0.013	Ti i (CH)	M
	10							210	23216.11	4306.142	4306.145	21.1		+ 0.003	CH	P
		10						280	23213.13	4306.695	4306.701	25.1		+ 0.006	CH	P
10								310	23212.33*	4306.844	4306.855	29.2		+ 0.011	CH	P
			9					180	23209.82	4307.309	4307.311	17.2		+ 0.002	CH	P
	9							300	23208.37	4307.578	4307.564	28.8		−0.014	CH?—CH	B
		9						200	23206.35	4307.953	4307.912	*165.7*	*s*	−0.041	|Fe i Ti ii (CH)	M
9								235	23205.20	4308.167	4308.176	16.7		+ 0.009	CH	P
			8					230	23203.01	4308.574	4308.595	25.1		+ 0.021	CH—CH	B
	8							230	23201.25	4308.901	4308.905	20.4		+ 0.004	CH	P
		8						260	23200.17*	4309.101	4309.131	13.2		+ 0.030	CH	P
								240	23198.68	4309.378	4309.383	29.9	*u*	+ 0.005	|Fe i CH	B
			7					220	23196.97	4309.695	4309.711	17.2	*w?*	+ 0.016	CH	P
	7	7						400	23194.83*	4310.903	4310.106	25.0		+ 0.013	CH	P
7								250	23192.87*	4310.457	4310.467	29.2		+ 0.010	CH	P
			6					200	23191.64*	4310.686	4310.704	22.7	*s*	+ 0.018	CH—	B
		6						220	23190.01	4310.989	4310.984	24.1		−0.005	CH	P
	6							300	23189.12*	4311.154	4311.167	17.9		+ 0.013	CH	P
6								} 320	$\{\;\begin{array}{c} 23187.62\;\\ 23187.27* \end{array}$	4311.433	4311.446	8.8		+ 0.013	CH	P
			5							4311.498	4311.509	39.6		+ 0.011	CH	P
		5						200	23186.06*	4311.723	4311.718	19.0		−0.005	CH	P
	5							} 280	$\{\;\begin{array}{c} 23184.15*\\ 23183.74* \end{array}$	4312.078	4312.086	23.0		+ 0.008	CH	P
			4							4312.155	4312.150	14.8		−0.005	CH	P
5		4						280	23183.00*	4312.292	4312.302	18.6	*w?d*	+ 0.010		B

Laboratory											Sun					

P_1dc_	P_1cd_	P_2dc_	P_2cd_	Q_1c_	Q_1d_	Q_2c_	Q_2d_	Intensity	Wave number cm^−1^	Wavelength A	Wavelength A	Disk int. Δλ/λ	Spot	⊙—lab. A	Solar identification	Notes
							3	141	23181.39*					−0.028	Mn i —CH	B
						3, 2	2	140	23180.78*	4312.706	4312.709	8.8		+ 0.003	CH	P
					4		‡	200?	23179.75*	4312.897	4312.875	35.5	*w?*	−0.022	|Ti ii—CH	B
				4			‡	200?	23179.05*	4313.027	4313.035	19.5	*wN*	+ 0.008	|CH Fe i p	B
							‡	M	23177.73	4313.273	4313.237	1.5		−0.036		A
							‡	} 160	$\{\;\begin{array}{c} 23177.15\\ 23176.04\\ 23175.66 \end{array}$	4313.381	4313.418	2.0		+ 0.037		A
					3					$\begin{array}{c} 4313.587\\ 4313.658 \end{array}\}$	4313.631	18.8		$\left\{\;\begin{array}{c} +0.044\\ -0.027 \end{array}\right\}$	CH—CH	
				3												B
							‡	6	23174.46*	4313.881	4313.890	2.6	*s*	+ 0.009		M
				2	2			124	23172.72*	4314.206	4314.221	7.9	*w*	+ 0.015	CH	P
			3					7	23094.44	4328.829	4328.845	2.1	*u*	+ 0.016	CH	P
		3						6	23093.98	4328.915	4328.927	1.3		+ 0.012	CH	P
							‡	20	$\{\;\begin{array}{c} 23092.75\\ 23092.26 \end{array}$	4329.146	4329.144	1.2		−0.002	CH	P
							‡			4329.238						A
	3							20	$\{\;\begin{array}{c} 23088.52\\ 23088.19 \end{array}$	4329.939						A
3										4330.001	4330.024	*8.1*	*s*	+ 0.023	V i	M
			4					18	23067.73	4333.842						A
		4						19	23066.89	4334.000	4334.018	2.3		+ 0.018	CH	P
							‡	10	23065.80	4334.204	4334.166	2.3		−0.038	Sm ii	M
	4							28	23063.39	4334.658	4334.672	3.9		+ 0.014	CH	P
4								28	23062.75	4334.778	4334.800	3.9	*s*	+ 0.022	CH—Ti i|	B
			5					40	23042.29*	4338.627	4338.627	3.2		0.000	CH	P
		5						40	23041.13	4338.845	4338.829	6.4	*ud*	−0.016	Cr i Fe i p	M
	5							45	23038.95	4339.256	4339.259	8.1	*u*	+ 0.003	Fe i (CH)	M
5								43	23037.92	4339.450	4339.456	27.4	*s*	+ 0.006	Cr i	M
			6					40	23018.16*	4343.175	4343.216	15.0	ud*	+ 0.041	|Cr i —Fe i p	M
		6						45	23016.49	4343.490	4343.494	10.4		+ 0.004	CH	P
	6							50	23015.42*	4343.692	4343.705	18.6	*u*	+ 0.013	Fe i (CH)	M
6								45	23013.97	4343.966	4343.968	11.5	*u*	+ 0.002	CH	P
			7					54	22995.12	4347.527	4347.545	9.0		+ 0.018	CH	P
	7	7						115	22992.82*	4347.961	4347.973	13.1		+ 0.012	CH	P
7								64	22990.84	4348.336	4348.338	12.0		+ 0.002	CH	P
			8					70	22972.96	4351.720	4351.710	0.5		−0.010	CH	P
	8							78	22971.08	4352.076	4352.072	16.8		−0.004	CH	P
		8						66	22970.14	4352.254	4352.261	12.2		+ 0.007	CH	P
8								77	22968.54	4352.558	4352.557	12.2		−0.001	CH	P
			9					70	22951.90	4355.713	4355.704	11.0		−0.009	CH	P
	9							78	22950.32	4356.013	4356.000	16.8		−0.013	CH	P
		9						77	22948.41	4356.375	4356.367	12.6		−0.008	CH	P
9								77	22947.14	4356.617	4356.604	12.4		−0.013	CH	P
			10					110	22931.91*	4359.510	4359.493	13.8		−0.017	CH	P
	10							80	22930.62	4359.755	4359.744	15.6	*w*	−0.011	Zr ii CH	B
		10						94	22927.76	4360.299	4360.289	13.3		−0.010	CH	P
10								94	22926.72	4360.497	4360.480	13.5	*s*	−0.017	Ti i CH	B
			11					100	22913.02	4363.105	4363.108	16.5	*ud*	+ 0.003	CH—Cr i	B
	11							103	22911.90	4363.318	4363.293	13.8	*wd*	−0.025	CH	P
		11						110	22908.07	4364.048	4364.041	11.0		−0.007	CH	P
11								110	22907.26	4364.202	4364.189	12.1		−0.013	CH	P
			12					100	22895.08*	4366.524	4366.500	14.4		−0.024	CH	P
	12							104	22894.13	4366.705	4366.675	16.5		−0.030	CH	P
		12						120	22889.32*	4367.622	4367.594	32.7	*u*	−0.028	|Fe i—CH	B
12								110	22888.68	4367.745	4367.723	3.2		−0.022	CH	P
			13					100	22878.29	4369.728	4369.714	6.2	*u*	−0.014	Ti i—Fe i p (CH)	M
	13							100	22877.55	4369.870	4369.866	2.3		−0.004	CH	P
		13						} 150	$\{\;\begin{array}{c} 22871.46\\ 22871.26 \end{array}$	4371.033	} 4371.062	17.8	*wd*	$\{\;\begin{array}{c} +0.029\\ -0.010 \end{array}$	} CH—CH	B
13										4371.072						
			14					100	22862.50	4372.746	4372.743	9.1		−0.003	CH	P
	14							100	22861.86	4372.869	4372.844	15.8		−0.025	CH	P
14		14						130	22854.73*	4374.233	4374.226	14.4		−0.007	CH	P
			15					90	22847.78	4375.563	4375.578	15.3		+ 0.015	CH	P
	15							90	22847.20	4375.675	4375.658	15.8		−0.017	CH	P
15		15						150	22838.96*	4377.253	4377.234	19.9		−0.019	CH	P
			16					} 100	$\{\;\begin{array}{c} 22833.79\\ 22833.50 \end{array}$	4378.244	} 4378.255	19.0		$\{\;\begin{array}{c} +0.011\\ -0.045 \end{array}$	} CH—CH?	B
	16									4378.300						
16		16						120	22824.21*	4380.282	4380.367	3.4	*u*	+ 0.085	Mg i (CH)	M
	17		17					125	22820.85*	4380.727	4380.724	21.0		−0.003	CH—	B
17								} 90	$\{\;$22810.49 22810.33	4382.716	4382.689	5.7		−0.027	CH	P
		17								4382.747	4382.764	22.4		+ 0.017	CH—Fe i|	B
	18		18					90	22808.95*	4383.012	4382.998	16.4		−0.014	—CH	B
	19		19					} 130	$\{\;\begin{array}{c} \;22798.05*\\ 22797.66\\ 22797.15 \end{array}$	4385.108	4385.124	14.1		+ 0.016	CH	P
18										4385.183						A
		18								4385.281	4385.254	12.1	*u*	−0.027	|Fe i—CH	B
	20		20					80	22787.78*	4387.084	4387.063	13.4		−0.021	CH	P
19								60	22785.64	4387.496	4387.497	13.9	*s*	+ 0.001	Cr i (CH)	M
		19						55	22785.10	4387.600	4387.595	9.6		−0.005	CH	P
	21		21					66	22778.38*	4388.894	4388.870	10.7		−0.024	CH	P
20								50	22774.41	4389.660	4389.638	10.2		−0.022	CH	P
		20						40	22773.73	4389.791	4389.776	6.8		−0.015	CH	P
	22		22					60	22769.83*	4390.543	4390.545	8.9		+ 0.002	CH	P
21								45	22763.95	4391.677	4391.668	11.8	*u?*	−0.009	Ce ii—CH	B
		21						44	22763.20	4391.821	4391.768	15.2	s	−0.053	Cr i (CH)	M
	23		23					44	22761.77*	4392.097	4392.071	12.3	*s*	−0.026	V i CH	B
22	24		24					60	22754.30*	4393.539	4393.524	16.2	*ud?*	−0.015	—CH	B
		22						35?	22753.39	4393.715	4393.700	6.4	*w*	−0.015	Fe i p—CH	B
	25		25					34	22747.36*	4394.879	4394.852	11.6	*s*	−0.027	Ti i	M
23								48	22745.11	4395.314	4395.289	3.4		−0.025	Fe i CH	B
		23						45	22744.16	4395.498	4395.504	15.0	*ud*	+ 0.006	CH—Fe i	B
	26							} 20	$\{\;\begin{array}{c} 22741.09\\ 22740.72 \end{array}$	4396.091	4396.079	5.0		−0.012	CH	P
			26							4396.163	4396.153	4.1		−0.010	—CH	B
24								36	22736.52	4396.975	4396.961	10.2		−0.014	CH	P
		24						} 22	$\{\;\begin{array}{c} 22735.60\\ 22734.82 \end{array}$	4397.153	4397.143	5.0	*u*	−0.010	CH	P
	27									4397.304						A
			27						22734.38	4397.389	4397.381	3.4	*u*	−0.008		M
25	28							24	22728.58*	4398.511	4398.491	7.7		−0.020	CH V ii	B
			28					16	22727.97	4398.629	4398.621	5.4	*w*	−0.008	Ni i CH	B
		25						14	22727.44	4398.732	4398.712	4.3	*w*	−0.020	CH	P
26								14	22720.92	4399.994	4399.989	4.8		−0.005	CH	P
		26						20	22719.74	4400.223	4400.185	9.3		−0.038	Ni i CH?	B
27									22713.45	4401.441	4401.451	15.0	*u*	+ 0.010	Fe i	M
		27							22712.19	4401.685	4401.668	2.5		−0.017	CH?	P
28									22706.19	4402.848	4402.841	4.1	*u*	−0.007	CH	P
		28							22704.86*	4403.106	4403.077	4.5	*uN*	−0.029	CH	P
29									22698.72	4404.297	4404.277	12.0	*u*	−0.020	|Ti i—CH	B
		29							22697.44	4404.546	4404.548	5.2		+ 0.002	CH?	P
30									22691.10	4405.776	4405.732	6.4	*sd*	−0.044	Ti i—CH?	B
		30							22689.73	4406.043	4406.036	3.0	*u*	−0.007	CH?	P
31									22683.17	4407.317						A
		31							22681.62	4407.618	4407.652	14.1	sd*	+ 0.034	V i	M
32									22674.32	4409.037						A
		32							22672.69	4409.354	4409.359	2.9	*uN*	+ 0.005		M
33									22664.71	4410.907						A
		33							22663.07*	4411.226	4411.227	5.4	*uN*	+ 0.001	CH—CH?	M
34									22654.44	4412.906	4412.877	1.4	*u*	−0.029		M
		34							22653.18	4413.152	4413.121	2.5		−0.031		M

* Blend.

‡ Satellite lines as follows:

**Table:** 

Designation	*J*	Laboratory	
		Wave number	Wavelength

R_Q 2d 1c_	3	23293.17*	4291.897
R_Q 2c 1d_ ; R_Q 2d 1c_	2; 2	23261.34*	4297.769
R_Q 2c 1d_ ; R_Q 2d 1c_	1; 1	23229.71*	4303.621
Q_R 1 2c_	3	23179.75*	4312.897
Q_R 1 2d_	2	23179.05*	4313.027
Q_P 2 1d_	3	23177.73	4313.273
Q_P 2 1c_	3	23177.15	4313.381
Q_P 2 1d_; Q_P 2 1c_	2; 2	23174.46*	4313.881
R_Q 1d 2c_	3	23092.75	4329.146
R_Q 1c 2d_	3	23092.26	4329.238
R_Q 1c 2d_	4	23065.80	4334.204

**Table 3:** CH in the Solar Spectrum A ^2^Δ—X ^2^Π (1, 1)

Laboratory											Sun					

Q_1c_	Q_1d_	Q_2c_	Q_2d_	R_1dc_	R_1cd_	R_2dc_	R_2cd_	Intensity	Wave number cm^−1^	Wavelength A	Wavelength A	Disk int. Δλ/λ	Spot	⊙–lab. A	Solar identification	Notes
				29, 28				3?	23886.86*	4185.222						A
						29, 28		4?	23883.64*	4185.787	4185.779	4.5		−0.008		M
				27				6?	23880.52	4186.334	4186.336	9.3	*uN*	+ 0.002	CN—Cr i	M
				30		27		M	23879.45*	4186.521						A
				26				4	23872.78	4187.691	4187.720	7.2		+ 0.029		M
						26		4	23871.58	4187.901	4187.812	20.8	*u*	−0.089	Fe i	M
				25				5	23861.81	4189.616	4189.565	16.7	*u*	−0.051	Fe i	M
						25		5	23860.75	4189.802	4189.816	3.8	*s*	+ 0.014	V i	M
				24	23		23	13	23848.12*	4192.021	4192.016	12.4	*u*	−0.005	CN?—CH	B
						24		6?	23847.29	4192.167	4192.204	3.6		+ 0.037	CH	P
				23				(CH)	23831.96*	4194.864	4194.848	15.5	*u*	−0.016	CH	M
						23		8?	23831.30	4194.980	4194.996	11.2	*sd?*	+ 0.016	CH Cr I—CN	B
					22		22	11	23828.76*	4195.427	4195.415	3.6		−0.012	Cr ii—CH	B
				22				10	23813.78	4198.066	4198.068	23.1	*uN*	+ 0.002	Fe i—	M
						22		10	23813.08	4198.189	4198.242	32.9	*u*	+ 0.053	Fe i	M
					21		21	15	23807.69*	4199.140	4199.105	43.6	*u*	−0.035	Fe i	M
				21				14	23793.81	4201.589	4201.577	7.4	*w*	−0.012	—CH	B
						21		12	23793.07	4201.720	4201.715	17.1	*w*	−0.005	|Ni i Fe i	M
					20		20	18	23785.01	4203.144	4203.129	9.5	*sd*	−0.015	—CH	B
				20				16	23772.11	4205.426	4205.390	9.8	*wN*	−0.036	Mn ii—CH	B
						20		14	23771.49	4205.535	4205.544	21.2	*s*	+ 0.009	Fe i (CH)	M
					19		19	16	23760.89*	4207.411	4207.408	14.0	*wN*	−0.003	CH	P
				19				19	23748.98	4209.521	4209.500	6.4	*u*	−0.021	CH	P
						19		18	23748.45	4209.615	4209.602	7.1	*u*	−0.013	CH	P
					18		18	33	23735.48*	4211.916	4211.895	15.4	*u*	−0.021	|Zr ii—CH	B
				18				} 26	$\{\;\begin{array}{c} 23724.56\\ 23724.27 \end{array}$	4213.854	4213.839	4.7	*u*	−0.015	CH	P
						18				4213.906	4213.909	10.7		+ 0.003	CH	P
					17		17	39	23709.04	4216.613	4316.599	11.6	*uN*	−0.014	CH	P
				17		17		} 36	$\{\;\begin{array}{c} 23699.07\\ 23698.85 \end{array}$	4218.387 4218.426	} 4218.392	12.6	*uN*	$\{\;\begin{array}{c} +0.005\\ -0.034 \end{array}$	} CH—CH	B
					16		16	43	23681.72*	4221.477	4221.469	12.8		−0.008	CH	P
				16		16		45	23672.66*	4223.093	4223.091	15.6		−0.002	CH	P
							15	} 45	$\{\;\begin{array}{c} 23653.77\\ 23653.58 \end{array}$	4226.465	4226.431	52.5	*u*	−0.034	Fe i	M
					15					4226.499						A
				15		15		54	23645.54*	4227.936	4227.944	15.6	*s*	+ 0.008	CH	P
							14	42	23625.14	4231.587	4231.609	9.7	*uN*	+ 0.022	CH—Zr ii	B
					14			40	23624.66	4231.673	4231.688	9.7	*uN*	+ 0.015	CH–Fe i	B
				14		14		63	23617.71*	4232.919	4232.927	15.1	*u*	+ 0.008	|CH—V i?	B
							13	40	23595.95	4236.822	4236.806	12.0	*u*	−0.016	CH V ii	B
					13			40	23595.30	4236.939	4236.958	7.8	*u*	+ 0.019	CH	P
				13		13		62	23589.28*	4238.020	4238.029	26.2	*s*	+ 0.009	Fe i	M
							12	46	23566.19	4242.172	4242.162	12.0		−0.010	Tm ii—CH|	B
					12			46?	23565.38*	4242.318	4242.283	5.2	wN*	−0.035	−CH	B
						12		45?	23560.54	4243.189	4243.203	14.8		+ 0.014	CH—	B
				12				45?	23560.12	4243.265						A
							11	41	23536.30	4247.561	4247.560	18.1	*wN*	−0.001	CH—	B
					11			45	23535.34	4247.734	4247.726	11.5		−0.008	CH	P
						11		48	23531.47	4248.432	4248.414	15.3	*s*	−0.018	|Fe I p—CH	B
				11				48	23530.89	4248.537	4248.534	9.4	*w*	−0.003	CH	P
							10	41	23506.18	4253.003	4253.004	8.9		+ 0.001	CH	P
					10			45	23505.04	4253.209	4253.210	9.4	*s*	+ 0.001	|CH	P
						10		46	23502.12	4253.738	4253.735	9.2		−0.003	CH	P
				10				50	23501.31	4253.885	4253.912	16.4	*sd*	+ 0.027	CH—Fe i	B
							9	42	23475.98	4258.475	4258.488	9.4	*u*	+ 0.013	CH—Ti i	B
					9			56	23474.60*	4258.725	4258.731	12.9	*w*	+ 0.006	CH—	B
						9		44	23472.61	4259.086	4259.098	9.4	*uN*	+ 0.012	CH	P
				9				48	23471.55	4259.278	4259.305	14.3	*sd?*	+ 0.027	V i Fe i (CH)	M
							8	40	23445.69	4263.976	4263.980	9.6	*w*	+ 0.004	CH	P
					8			44	23444.06	4264.273	4264.281	2.3	*U*	+ 0.008	CH	P
						8		42	23442.96	4264.473	4264.468	13.4	*s*	−0.005	CH	P
				8				46	23441.63	4264.715	4264.741	20.2	*U*	+ 0.026	Fe i (CH)	M
							7	44	23415.51	4269.472	4269.478	9.1	*w*	+ 0.006	CH—La ii	B
					7	7		82	23413.48*	4269.842	4269.849	17.8	*U*	+ 0.007	CH—Fe i p	B
				7				46	23411.70	4270.167	4270.170	15.0	*sd*	+ 0.003	|Ti i CH	B
							6	42	23385.46	4274.958	4274.959	20.1	*uN*	+ 0.001	CH	P
						6		80	23383.88*	4275.247	4275.258	15.0		+ 0.011	CH	P
					6			104	23383.17*	4275.377	4275.386	24.3		+ 0.009	CH	P
				6				50	23381.73	4275.640	4275.661	10.5	ud*	+ 0.021	CH La ii	B
							5	50	23355.75*	4280.396	4280.403	14.2	*w*	+ 0.007	Cr i CH	B
						5		60	23354.59	4280.609	4280.632	7.2		+ 0.023	Fe i p CH	B
					5			60	23352.70*	4280.956	4280.964	11.4		+ 0.008	CH	P
				5				50	23351.87	4281.108	4281.100	18.0	*s*	−0.008	|Mn i—CH	B
							4	30	23326.34	4285.794	4285.815	13.8	*s*	+ 0.021	Co i—Fe i (CH)	M
						4		40	23325.56	4285.937	4285.938	1.8		+ 0.001	CH	P
					4			(CH)	23322.56*	4286.488	4286.477	26.6	*uN*	−0.011	Fe i—CH	M
				4				M	23322.08	4286.576	4286.587	11.2	*uN*	+ 0.011	CH	P
							3	(CH)	23297.42*	4291.113	4291.121	26.1	*U*	+ 0.008	CH	M
						3		(CH)	23296.85*	4291.219	4291.220	14.2	*u*	+ 0.001	CH Ti i	M
							‡	(CH)	23293.17*	4291.897	4291.839	2.6	*sd*	−0.058	V i—	M
					3			M	23292.69	4291.985	4291.979	6.5	*w*	−0.006	Cr i—CH	B
				3				(CH)	23292.36*	4292.046	4292.055	12.3		+ 0.009	CH	M
						2	2	56	23269.80*	4296.207	4296.217	8.1		+ 0.010	CH	P
							‡	(CH)	23263.96*	4297.286	4297.291	20.9		+ 0.005	CH	M
				2	2			120	23262.70*	4297.518	4297.530	17.4	*wd?*	+ 0.012	CH—	B
	19		19					(CH)	23253.34*	4299.248	4299.249	49.3	*sN*	+ 0.001	|Fe i Ti I (CH)	M
						1	1	} 80	$\{\;\begin{array}{c} 23252.00*\\ 23249.13* \end{array}$	4299.496	4299.483	18.1	*u*	−0.013	CH Fe i p	B
	18		18							4300.026	4300.053	38.6	*w*	+ 0.027	Ti ii	M
			17					M	23245.09	4300.774	4300.744	11.8	*uN*	−0.030		M
	17							M	23244.61	4300.863	4300.827	23.0	*u*	−0.036	Fe i	M
19		19						(CH)	23243.12*	4301.138	4301.103	41.6	*s*	−0.035	Ti i (V ii)	M
			16					} 88	$\{\;\begin{array}{c} 23239.89\\ 23239.69 \end{array}$	4301.736	} 4301.749	15.1		$\{\;\begin{array}{c} +0.013\\ -0.024 \end{array}$	} CH—CH	B
	16									4301.773						
18		18						50?	23239.22*	4301.860	4301.927	34.4	*u*	+ 0.067	Ti ii	M
17		17						M	23235.12*	4302.619	4302.539	38.4	*S*	−0.080	Ca i	M
			15					M	23234.75	4302.688	4302.65	19.5		−0.04		M
	15						‡	(CH)	23234.38*	4302.756	4302.754	24.2		−0.002	CH	M
				1	1			68	23232.47*	4303.110	4303.089	9.3	*w?N*	−0.021	CH	P
16		16						88	23230.85*	4303.410	4303.426	16.7		+ 0.016	CH	P
			14					M	23229.30*	4303.697	4303.723	13.2	*w*	+ 0.026	CH—	B
	14							(CH)	23228.59*	4303.829	4303.835	28.6	*u*	+ 0.006	CH	M
15		15						M	23226.26*	4304.261	4304.256	14.6	*w*	−0.005	CH	P
			13					M	23223.84	4304.709	4304.721	9.0		+ 0.012	CH	P
	13							M	23223.09	4304.848	4304.852	13.0	*uN*	+ 0.004	CH—Fe i	B
14		14						88	23221.71*	4305.104	4305.110	19.0		+ 0.006	CH Fe i	B
			12					48	23218.55	4305.690	4305.713	15.6	*u*	+ 0.023	Sc ii	M
	12							M	23217.78	4305.833	4305.847	5.8		+ 0.014	CH	P
13		13						(CH)	23217.08*	4305.962	4305.918	36.2	*S*	−0.044	Ti i (CH)	M
			11					M	23213.41	4306.643	4306.599	12.5	*uN*	−0.044	Fe i	M
		12						M	23212.73	4306.769						A
12	11							(CH)	23212.33*	4306.844	4306.855	29.2		+ 0.011	CH	M
			10					M	23208.67	4307.523	} 4307.564	28.8		$\{\;\begin{array}{c} +0.041\\ -0.014 \end{array}$	} CH?—CH	B
		11						(CH)	23208.37*	4307.578						M
11	10							M	23207.52*	4307.736	4307.748	40.2	*s*	+0.012	Ca i	M
		10						M	23204.74	4308.252	4308.289	12.8		+0.037		A
			9					M	23204.18*	4308.356	4308.381	14.2		+ 0.025	CH	P

Laboratory											Sun					

P_1dc_	P_1cd_	P_2dc_	P_2cd_	Q_1c_	Q_1d_	Q_2c_	Q_2d_	Intensity	Wave number cm^−1^	Wavelength A	Wave length A	Disk int. Δλ/λ	Spot	⊙—lab. A	Solar identification	Notes
				10				M	23203.77*	4308.432	4308.441	11.1		+ 0.009	CH	P
					9			M	23202.76	4308.620	4308.595	25.1		−0.025	CH—CH	B
						9		M	23200.84	4308.976	4309.040	31.6	*u*	+ 0.064	Fe i	M
							8	(CH)	23200.17*	4309.100	4309.130	13.2		+ 0.030	CH	M
				9				M	23199.74	4309.181	4309.205	13.2		+ 0.024	CH	P
					8			M	23198.31	4309.446	4309.458	21.6	*w*	+ 0.012	CH Fe i p	B
						8		M	23197.31*	4309.632	4309.634	21.8	*u*	+ 0.002	‖Y ii CH	B
							7	M	23196.30	4309.820	4309.834	9.5	*sN*	+ 0.014	CH V i	B
				8				M	23195.88	4309.898	4309.900	16.5	*uN*	+ 0.002	CH	P
					7	7		M	23194.19*	4310.212	4310.225	18.3		+ 0.013	CH	P
							6	(CH)	23192.87*	4310.457	4310.467	29.2		+ 0.010	CH	M
				7				M	23192.41	4310.543	4310.559	9.3		+ 0.016	CH	P
						6		(CH)	23191.64*	4310.686	4310.704	22.7	*s*	+ 0.018	CH—	M
					6		5	M	23190.56*	4310.886	4310.897	10.0		+ 0.011	CH	P
				6		5		(CH)	23189.12*	4311.154	4311.167	17.9		+ 0.013	CH	M
							4	M	23188.26	4311.314	4311.322	5.6		+ 0.008	CH	P
					5	4		(CH)	23187.27*	4311.498	4311.509	39.6		+ 0.011	CH	M
				5		3, 2	3, 2	(CH)	23186.06*	4311.723	4311.718	19.0		−0.005	CH	M
							‡	M	23185.33*	4311.859	4311.888	4.4	*u*	+ 0.029		M
					4		‡	(CH)	23184.15*	4312.078	4312.086	23.0		+ 0.008	CH	M
				4				(CH)	23183.74*	4312.155	4312.150	14.8		−0.005	CH	M
					3		‡	M	23181.72*	4312.531	4312.504	4.6	*u*	−0.027	Cr i?—CH	B
				3				(CH)	23181.39*	4312.592	4312.564	20.9		−0.028	Mn i—CH	M
							‡	(CH)	23179.75*	4312.897	4312.875	35.5	*w?*	−0.022	|Ti ii—CH	M
				2	2			(CH)	23179.05*	4313.027	4313.035	19.5	*wN*	+ 0.008	|CH Fe i p	M
			3					} 24	$\{\;\begin{array}{c} 23104.00\;\\ 23103.46* \end{array}$	4327.038	4327.110	18.2	*w*	+ 0.072	Fe i	M
		3								4327.139	4327.157	2.0	*u?*	+ 0.018	CH	P
							‡	8	23102.23	4327.370	4327.317	1.4		−0.053		A
							‡	8	23101.32	4327.540						A
	3							6	23098.25	4328.115						A
3								6	23097.89	4328.183	4328.203	2.0		+ 0.020	CH?	P
			4					20	23077.53	4332.001	4332.006	1.2	*o*	+ 0.005	CH—	B
		4						14	23076.75	4332.148	} 4332.169	1.0		$\{\;\begin{array}{c} +0.021\\ -0.037 \end{array}$	CH	P
							‡		23076.44	4332.206						A
							‡		23075.60	4332.364						A
	4							8	23073.37	4332.783	4332.831	4.8	*s*	+ 0.048	V i	A
4								12	23072.71	4332.906	4332.918	8.3	*u*	+ 0.012	CH—	B
			5					20	23052.10	4336.780	4336.791	2.3	*u*	+ 0.011	CH	P
		5						14	23050.92	4337.002	4337.055	30.4	*U*	+ 0.053	Fe i	M
							‡	M	23050.18	4337.142						A
	5							20	23048.79	4337.403	4337.411	4.8	*u*	+ 0.008	CH	P
5								20	23047.87	4337.576	4337.566	23.3	*s*	−0.010	Cr i	M
			6					20	23027.55	4341.404	4341.371	19.6	*w?*	−0.033	Ti ii	M
							‡	20?	23026.97	4341.513	4341.551	4.8	*uN*	+ 0.038	Fe i p	A
		6						20	23025.95	4341.705	4341.710	5.8	*w*	+ 0.005	CH	P
	G							20	23024.93	4341.898	4341.924	4.8	*u?*	+ 0.026	CH	P
6								20	23023.50	4342.168	4342.180	6.4	*U*	+ 0.012	Gd ii CH	B
			7					18	23003.77	4345.892	4345.895	5.8	*u*	+ 0.003	CH	P
	7	7						43	23001.62*	4346.298	4346.295	12.0	*U*	−0.003	CH	P
7								22	22999.68	4346.665	4346.673	3.7	*u*	+ 0.008	CH	P
			8					21	22980.73	4350.249	4350.249	6.4	*u*	0.000	CH	P
	8							22	22978.93	4350.589	4359.585	8.3	*u*	−0.004	Fe i—CH	B
		8						21	22978.01	4350.764	4350.760	3.9		−0.004	CH	P
8								26	22976.47	4351.055	4351.056	*20.0*	*s*	+ 0.001	Cr i	M
			9					20	22958.39	4354.482	4354.514	8.3	uNN*	+ 0.032	Mg i	M
	9							18	22956.88	4354.768	4354.762	5.7		−0.006	CH Fe i?	B
		9						28	22955.02	4355.121	4355.093	*23.0*	*s*	−0.028	Ca i (CH)	M
9								24	22953.82	4355.349	4355.351	8.0	*u*	+ 0.002	CH	P
			10					20	22936.77	4358.586	4358.512	21.8	*u*	−0.074	Fe i	M
	10							36	22935.49	4358.830	4358.820	15.6	*uN*	−0.010	—CH	P
		10						30?	22932.73	4359.354	4359.341	6.6		−0.013	CH	B
10								110	22931.91*	4359.510	4459.493	13.8		−0.017	CH	P
			11					28	22915.87*	4362.562	4362.533	12.1	*w?*	−0.029	—CH	B
	11							32	22914.81*	4362.764	4362.746	10.3	*u*	−0.018	CH	P
		11						40?	22911.09	4363.473	4363.467	5.5	*u*	−0.006	CH	P
11								30?	22910.33	4363.617	4363.602	6.2	*w*	−0.015	Mo ii—CH	B
			12					M	22895.53	4366.438	4366.413	5.5	*uN*	−0.025	—CH	B
	12							(CH)	22895.08*	4366.524	4366.500	14.4		−0.024	CH	M
		12						M	22889.92*	4367.508	4367.475	5.7	*u*	−0.033	CH	P
12								(CH)	22889.32*	4367.622	4367.594	32.7	*u*	−0.028	|Fe i—CH	M
			13					28	22876.01	4370.164	4370.154	6.2		−0.010	CH	P
	13							32	22875.25	4370.309	4370.292	5.9		−0.017	CH	P
		13						} 50?	$\{\;\begin{array}{c} 22869.46\\ 22869.24 \end{array}$	4371.415	} 4371.426	11.9	*wd?*	$\{\;\begin{array}{c} +0.011\\ -0.031 \end{array}$	} CH—CH	
13										4371.457						B
			14					38	22857.00	4373.799	4373.791	7.8	*u*	−0.008	CH	P
	14							34?	22856.50	4373.894	4373.888	7.8		0.006	CH Fe i p	B
14		14						48	22849.59*	4375.217	4375.202	12.6		−0.015	CH	P
			15					(CH)	22838.96*	4377.253	4377.234	19.9		−0.019	CH	M
	15							M	22838.10	4377.418	4377.374	8.4	*u*	−0.044	Fe i—CH	B
15		15						48	22830.26*	4378.921	4378.913	10.0		−0.008	CH	P
			16					(CH)	22820.85*	4380.727	4380.724	21.0		−0.003	CH—	M
	16							M	22820.27*	4380.838	4380.852	9.6	*wN*	+ 0.014	CH—	B
16		16						40	22811.40*	4382.542	4382.521	8.2		−0.021	CH	P
			17					} 32	$\{\;\begin{array}{c} 22803.22\\ 22803.00 \end{array}$	4384.114	} 4384.120	8.4		$\{\;\begin{array}{c} +0.006\\ -0.036 \end{array}$	CH—CH	B
	17									4384.156						
17		17						31	22792.92*	4386.095	4386.063	9.8		−0.032	—CH	B
	18		18					M	22786.09*	4387.410	4387.398	10.5	*u*	−0.012	—CH	B
18		18						30?	22775.14*	4389.519	4389.504	4.6		−0.015	CH	P
	19		19					M	22769.35*	4390.635	4390.630	7.5	*u*	−0.005	CH	P
19								18?	22757.45	4392.931	4392.924	4.8	*u*	−0.007	CH	P
		19						18?	22756.89	4393.039	4393.025	6.1	*s?*	−0.014	CH F ip	B
	20		20					15?	22752.83*	4393.823	4393.808	6.4	*u*	−0.015	CH V i	B
20								28?	22739.85*	4396.331	4396.309	8.2		−0.022	CH	P
		20						20?	22739.39	4396.420	4396.430	4.1	*u*	+ 0.010	CH	P
	21		21					(CH)	22736.52*	4396.975	4396.961	10.2		−0.014	CH	M
21									22722.42	4399.704	} 4399.778	*26.4*	*w?*	$\{\;\begin{array}{c} +0.074\\ -0.061 \end{array}$	} Ti ii (Cr i)	M
		21							22721.72	4399.839						
	22		22						22719.74*	4400.223	4400.185	9.3		−0.038	Ni i CH?	M
22								(CH)	22704.86*	4403.106	4403.077	4.5	*uN*	−0.029	CH	M
		22						(CH)	22704.16	4403.242	4403.187	14.5	*u*	−0.055		A
	23		23						22703.21*	4403.426	4403.374	10.7	*s*	−0.052	‖Cr i Zr ii	A
23									22687.27	4406.521	4406.504	4.5	*u*	−0.017	CH	P
	24	23	24						22686.36*	4406.696	4406.652	*18.2*	*S*	−0.044	V i	M
24	25		25						22669.17*	4410.039	4410.014	5.7		−0.025	—CH?	B
		24							22668.37	4410.195	4410.167	5.7		−0.028	—CH?	B
25									22650.69	4413.637	4413.599	8.8	*w*	−0.038	|Fe i CH?	B
		25							22649.72	4413.826	4413.853	6.6	*u*	+ 0.027	Cr i	M
26									22631.45	4417.390	4417.412	3.4	*s*	+ 0.022	Co i	M
		26							22630.37	4417.600	4417.574	2.5	*u*	−0.026	CH	P
27									22611.22	4421.342	4421.334	4.8	*u*	−0.008	Co i—CH	B
		27							22610.08	4421.565	4421.573	7.9	*S*	+ 0.008	V i	M
28									22589.60	4425.573						A
		28							22588.33	4425.822	4425.769	5.0	*uN*	−0.053	Fe i p—Fe i p	A
29									22566.49	4430.105						A
		29							22565.28	4430.343	4430.368	6.1	*s*	+ 0.025	Ti i	M
30									22541.17	4435.081						A
		30							22539.67	4435.376	4435.328	5.9	*u*	−0.048	Ni i?	A
31									22513.21	4440.589	4440.630	4.3		+ 0.041	Fe i	A
		31							22512.03	4440.823	4440.827	*10.4*	*u*	+ 0.004		M
32									22482.25	4446.705						A

* Blend.

‡ Satellite lines as follows:

**Table:** 

Designation	*J*	Laboratory	
		Wave number	Wavelength

R_Q 2c 1cd_ ; R_Q 2d 1c_	3; 3	23293.17*	4291.897
B_Q2d 1c_	2	23263.96*	4297.286
B_Q 2d 1c_	1	23234.38*	4302.756
Q_P2 1d_ ; Q_R1 2d_ ; Q_R1 2c_	4; 2; 2, 3	23185.33*	4311.859
Q_P2 1c_	4	23184.15*	4312.078
Q_P2 1d_	3	23181.72*	4312.531
Q_P2 1d;_ Q_P2 1c_	2; 2	23179.75*	4312.897
P_Q 1c 2d_	3	23102.23	4327.370
P_Q 1d 2c_	3	23101.32	4327.540
P_Q 1c 2d_	4	23076.44	4332.206
P_Q 1d 2c_	4	23075.60	4332.364
P_Q 1d 2c_	5	23050.18	4337.142
P_Q 1c 2d_	6	23026.97	4341.513

**Table 4:** CH in the Solar Spectrum A ^2^Δ—X ^2^Π (2, 2)

Laboratory											Sun					

Q_1c_	Q_1d_	Q_2c_	Q_2d_	R_1dc_	R_1cd_	R_2dc_	R_2cd_	Intensity	Wave number cm^−1^	Wavelength A	Wavelength A	Disk int. Δλ/λ	Spot	⊙—lab. A	Solar identification	Notes
					20		20	7	23587.31^*^	4238.374	4238.395	9.7	*ud?*	+ 0.021	CH La ii	B
				23, 22				6	23584.39^*^	4238.899	4238.944	9.7	*sN*	+ 0.045	Cr i	M
						22		4	23583.67	4239.028	4239.044	3.8		+ 0.016	Fe i p CH	B
						23		3	23583.23	4239.108	4239.145	2.4		+ 0.037		A
				21				} 5	$\{\;\begin{array}{c} 23581.18\\ 23580.53 \end{array}$	4239.476	4239.485	2.1	*u*	+ 0.009	CH	P
						21				4239.592	4239.599	2.6	*u*	+ 0.007	CH	P
				24				2	23579.75	4239.733	4239.733	19.8	*u*	0.000	Mn i—Fe i	M
						24		2	23578.80	4239.904	4239.953	16.5	*u*	+ 0.049	Fe i	M
					19		19	8	23577.18^*^	4240.195	4240.199	4.7	*w?*	+ 0.004	CH	P
				20				6	23574.93	4240.600	4240.610	2.2		+ 0.010	CH	P
						20		6	23574.29	4240.715	4240.702	14.1	*u*	−0.013	Cr i	M
				25				2	23570.94	4241.318	4241.328	1.2	*u*	+ 0.010	CH—-	B
						25		2	23569.86	4241.512	4241.521	2.8	*sd*	+ 0.009	CH	B
				19				(CH)	23565.38^*^	4242.318	4242.283	5.2	wN^*^	−0.035	—CH	M
					18	19	18	(CH)	23564.69^*^	4242.443	4242.455	20.0		+ 0.012	CH	M
				18				} 9	$\{\;\begin{array}{c} 23554.22\\ 23553.81 \end{array}$	4244.328	4244.340	3.1	*u*	+ 0.012	CH	P
						18				4244.403	4244.403	3.3	*u*	0.000	CH—Fe i	B
					17		17	12	23550.06^*^	4245.078	4245.082	6.6	*wN*	+ 0.004	CH—	B
				17				} 6	$\{\;\begin{array}{c} 23540.65\\ 23540.32 \end{array}$	4246.776	} 4246.837	40.3	*w?*	$\{\begin{array}{c} +0.061\\ +0.002 \end{array}$	} Sc ii	M
						17				4246.835						
					16		16	11	23533.52^*^	4248.062	4248.055	7.3	*w?N*	−0.007	CH	P
				16				(CH)	23524.74^*^	4249.648	4249.637	23.0	*ud*	−0.01l	—CH	M
						16		M	23524.50	4249.691					—CH	A
					15		15	13	23515.35^*^	4251.345	4251.331	8.2	*s*	−0.014		B
				15		15		18	23507.64^*^	4252.739	4252.756	11.8	*u*	+ 0.017	CH	P
							14	M	23496.01	4254.844	4254.846	3.3	*u*	+ 0.002	CH	P
					14			(CH)	23495.17^*^	4254.996	4254.979	27.0	*ud*	−0.017	Fe i—CH	M
				14		14		24	23488.93^*^	4256.127	4256.136	5.2	*0*	+ 0.009	CH—	B
							13	M	23475.27	4258.603	4258.619	19.2	*u*	+ 0.016	Fe i	M
					13			(CH)	23474.60^*^	4258.725	4258.731	12.9	*w*	+ 0.006	CH—	M
				13		13		22	23468.90^*^	4259.759	4259.764	8.2	*w?*	+ 0.005	CH—	B
							12	15	23453.31	4262.591	4262.585	4.7	*w*	−0.006	CH	P
					12			15	23452.66	4262.709	4262.710	7.3		+ 0.001	CH	P
						12		} 20	$\{\begin{array}{c} 23447.98\\ 23447.58 \end{array}$	4263.560	} 4263.608			$\{\begin{array}{c} +0.048\\ -0.024 \end{array}$	} CH—CH	B
				12						4263.632		8.7	*ud?*			
							11	(CH)	23430.54^*^	4266.733	4266.742	10.3		+ 0.009	CH	M
					11			16?	23429.67	4266.891	4266.968	19.0	*u*	+ 0.077	Fe i	M
						11		M	23425.88	4267.582	4267.588	4.9	*s*	+ 0.006	CH	P
				11				(CH)	23424.91^*^	4267.759	4267.749	13.1	*uN*	−0.010	CH	M
							10	10	23406.90	4271.042	4271.057	6.1		+ 0.015	CH—Cr i	B
					10			10	23405.76	4271.250	4271.164	52.2	*u*	−0.086	Fe i	M
						10		10	23402.96	4271.761	4271.774	177.0	*s*	+ 0.013	Fe i	M
				10				10	23402.13	4271.913	4271.949	18.2	*uN*	+ 0.036	Fe i p CH	B
							9	8?	23382.42	4275.514	4275.513	2.6		−0.001	CH	P
					9			8?	23380.90	4275.791						A
						9		8?	23379.27	4276.090	4276.103	4.7		+ 0.013	CH—	B
				9					23378.17	4276.291	4276.274	4.4		−0.017	—CH	B
					8		8	(CH)	23357.76^*^	4280.028	4280.037	15.0	*wd?*	+ 0.009	CH	M
						8		(CH)	23355.75^*^	4280.396	4280.403	14.2	*w*	+ 0.007	Cr i CH	M
								M	23355.21	4280.495	4280.494	2.8	s^*^	−0.001	Gd ii	M
				8				(CH)	23353.65	4280.781	4280.788	13.7		+ 0.007	Sm ii CH	M
							7	10?	23332.39	4284.683	4284.686	11.7	*u*	+ 0.003	Ni i	M
					7	7		M	23330.41^*^	4285.046	4285.008	17.5	*s*	−0.038	CH Ti i|	M
				7				(CH)	23328.63^*^	4285.373	4285.371	7.9		−0.002	CH	M
							6	14	23306.74	4289.398	4289.372	30.5	*s*	−0.026	Ca i	M
						6		14	23305.26	4289.670	} 4289.729	53.6	*S*	$\{\begin{array}{c} +0.059\\ -0.086 \end{array}$	} Cr i	M
					6			14	23304.47	4289.815						
				6				14	23303.01	4290.084	4290.057	4.9	*u*	−0.027	CH	P
							5	14	23280.93	4294.153	4294.142	50.5	*s*	−0.011	Fe i Ti ii	M
						5		14	23279.84	4294.354	4294.370	6.3	*u*	+ 0.016	CH	P
					5			16	23278.19	4294.659	4294.623	7.2	*ud*	−0.036	W i—CH	B
				5				16	23277.23	4294.836	4294.859	1.6		+ 0.023	CH	P
							4	M	23254.96	4298.949	4298.994	27.2	*s*	+ 0.045	Ca i	M
						4		(CH)	23253.88^*^	4299.148	4299.138	24.2	*u?*	−0.010	CH	M
					4			M	23251.70	4299.551	4299.483	18.1	*u*	−0.068	CH Fe i p	M
				*4*				M	23250.98	4299.684	4299.645	19.1	*s*	−0.039	‖Ti i Fe i	M
							3	(CH)	23229.30^*^	4303.697	4303.723	13.2	*W*	+ 0.026	CH—	M
						3		(CH)	23228.59^*^	4303.829	4303.835	28.6	*u*	+ 0.006	CH	M
				3	3			(CH)	23224.53^*^	4304.581	4304.571	25.1	*w*	−0.010	Fe i CH	M
							2	(CH)	23204.18^*^	4308.356	4308.381	14.2		+ 0.025	CH	M
								(CH)	23203.77^*^	4308.432	4308.441	11.1		+ 0.009	CH	M
				2	2			(CH)	23197.31^*^	4309.632	4309.634	21.8	*u*	+ 0.002	‖Y ii CH	M
						1	1	(CH)	23189.12^*^	4311.154	4311.167	17.9		+ 0.013	CH	M
				1	1			28	23169.49^*^	4314.807	4314.807	16.2	*s*	0.000	Ti i	M
			11, 10, 9, 8					} 100	{23125.56^*^	4323.004	4323.013	15.5		+ 0.009	CH	P

Laboratory											Sun					

P_1dc_	P_1cd_	P_2dc_	P_2cd_	Q_1c_	Q_1d_	Q_2c_	Q_2d_	Intensity	Wave Number ber cm^−1^	Wavelength A	Wavelength A	Disk int. Δλ/λ	Spot	⊙—lab. A	Solar identification	Notes
						2	12, 7, 2	}	{23125.26^*^	4323.059	4323.063	6.5		+ 0.004	CH	P
					12, 11, 10 13, 9		13, 6, 5 4	} 150	$\{\begin{array}{c} 23124.49*\\ 23124.26* \end{array}$	4323.204 4323.247	} 4323.226	23.6		$\{\begin{array}{c} +0.022\\ -0.021 \end{array}$	} CH—CH	B
					8		14, 3	90	23123.67^*^	4323.357	4323.367	11.1	*u*	+0.010	CH Fe i p	B
					14, 7	$\left\{\begin{array}{l} 9,8,7\\ 6,5,4\\ 3 \end{array}\right\}$		} 120	$\{\begin{array}{c} 23122.98*\\ 23122.25* \end{array}$	4323.487	4323.512	24.7	*u*	+ 0.025	CH—	B
					6	10				4323.622	4323.611	18.7	*uN*	−0.011	CH	P
				9, 8, 7, 6	5	11	15	} 155	$\{\begin{array}{c} 23121.14*\\ 23120.47*\\ 23119.78* \end{array}$	4323.830	4323.851	26.6		+ 0.021	CH	P
				10, 5	15, 4					4323.955	4323.973	13.2		+ 0.018	CH	P
				11, 4		12				4324.084	4324.087	9.9		+ 0.003	CH	P
				12, 3	3			60	23119.23^*^	4324.188	4324.176	9.2		−0.012	CH	P
				13	16	13	16	} 60	$\{\begin{array}{c} 23118.20*\\ 23118.04* \end{array}$	4324.381	} 4324.412	17.8		$\{\begin{array}{c} +0.031\\ +0.002 \end{array}$	} CH CH	B
				2	2					4324.410						
				14		14		31	23115.79^*^	4324.831	4324.819	9.9	*w*	−0.012	—CH	B
					17		17	25	23114.36^*^	4325.099	4325.141	16.6	*s*	+ 0.042	Ti i	M
				15		15		28	23113.03^*^	4325.348	4325.356	15.5	*u*	+ 0.008	CH	P
				16	18	16	18	43	23109.38^*^	4326.031	4326.052	13.2	*uN*	+ 0.021	CH	P
				17				20	23104.87	4326.875	} 4326.917	8.6	*w*	$\{\begin{array}{c} +0.042\\ -0.020 \end{array}$	} CH—CH	B
						17		18	23104.54	4326.937						
					19		19	20	23103.46^*^	4327.139	4327.157	2.0	*u?*	+ 0.018	CH	P
				18				} 14	$\{\begin{array}{c} 23099.13\\ 23098.73 \end{array}$	4327.951	4327.917	16.2	*u*	−0.034	Fe i	M
						18				4328.025	4328.035	6.2		+ 0.010	CH	P
					20		20	15	23095.65^*^	4328.602	4328.610	8.1	*u*	+ 0.008	CH	P
				19				} 20	$\{\begin{array}{c} 23092.07\\ 23091.55 \end{array}$	4329.274	4329.289	6.9	*u*	+ 0.015	CH	P
						19				4329.371	4329.391	6.0	*u*	+ 0.020	CH	P
					21		21	12	23086.07^*^	4330.399	4330.408	5.3	uN^*^	+ 0.009	CH	P
				20				4	23083.08	4330.960	4330.956	9.7	*u*	−0.004	Fe i	M
						20		10	23082.71	4331.029	4331.053	3.5	*w*	+ 0.024	CH—	B
					22		22	10	23074.38^*^	4332.593	4332.583	6.7	*uN*	−0.010	Cr i—CH	B
				21				} 10	$\{\begin{array}{c} 23071.89\\ 23071.36 \end{array}$	4333.061	4333.051	2.8	*u*	−0.010	CH Fe i p	B
						21				4333.160	4333.206	3.2	*u*	+ 0.046	Ni i? CH?	B
					23		23	12	23060.07^*^	4335.282	4335.274	7.6	*u*	−0.008	CH	P
			3					3?	23046.10	4337.909	4337.925	24.0	*w*	+ 0.016	Ti ii	M
		3						M	23045.62	4338.000						A
					24		24	8?	23042.30^*^	4338.625	4338.627	3.2		+ 0.002	CH	P
3	3							4	23040.32^*^	4338.998	4339.010	2.3	*u*	+ 0.012	CH	P
					25			6	23020.97	4342.645						A
							25	2	23020.54	4342.726						A
			4					4?	23019.34	4342.952	4342.990	1.7	*u*?	+ 0.038	CH?	P
		4						(CH)	23018.16^*^	4343.175	4343.216	15.0	ud^*^	+ 0.041	Cr i Fe i p	M
	4							(CH)	23015.42^*^	4343.692	4343.705	18.6	*u*	+ 0.013	Fe i	M
4								M	23014.66	4343.835	4343.854	0.8		+ 0.019	CH—Fe i p	B
					26			4?	22996.59	4347.249	4347.242	9.9	*s*	−0.007	Fe i	M
							26	4?	22995.86	4347.386	4347.370	0.8		−0.016	—CH	B
			5					M	22993.38	4347.855	4347.841	15.4	*w?*	−0.014	Fe i	M
		5						M	22992.02	4348.113	4348.104	2.5		−0.009	CH	P
	5							M	22990.15	4348.466	4348.492	2.3	*u*	+ 0.026	CH	P
5								4	22989.26	4348.635	4348.636	1.7	*u*	+0.001	CH	P
			6					4	22967.77	4352.704	4352.743	32.6	*s*	+ 0.039	Fe i	M
		6						} 2	$\{\begin{array}{c} 22965.78\\ 22965.27 \end{array}$	4353.081	4353.053	2.5		−0.028	CH	P
	6									4353.177	4353.172	6.9	*o?*	−0.005	—CH	B
6								3	22963.88	4353.441	4353.435	2.3	*uN*	— 0.006	CH	P
			7					4	22942.48	4357.502	4357.514	8.7		+0.012	Cr i Fe i pFe i p	M
	7	7						7	22940.47^*^	4357.883	4357.869	5.5	*u*	−0.014	CH	P
7								3	22938.68	4358.224	4358.170	8.7	*s*	−0.054	Nd ii	M
			8						22917.66	4362.221	4362.216	5.5	*u*	−0.005	CH	P
	8								22915.87^*^	4362.562	4362.533	12.1	*w?*	−0.029	—CH	M
		8							22914.81^*^	4362.764	4362.746	10.3	*u*	−0.018	CH	M
8									22913.71	4362.974	4362.953	3.9	*u*	−0.021	Cr i Cr ii	M
			9						22892.74	4366.970						A
	9								22891.49	4367.208	4367.195	2.5		−0.013	Ni i—CH	B
		9							22889.92^*^	4367.508	4367.475	5.7	*u*	−0.033	CH	P
9									22888.68^*^	4367.745	4367.723	3.2		−0.022	CH	M
			10						22868.46	4371.606	4371.582	2.7		−0.024	CH	P
	10								22867.24	4371.840	4371.794	4.1	*u*	−0.046		M
		10							22864.72	4372.322	4372.335	6.2	sd^*^	+ 0.013	CH—	B
10									22863.79	4372.500	4372.493	3.0		−0.007	CH	P
			11						22844.21	4376.247	4376.216	5.2		−0.031	CH	P
	11								22843.32	4376.418	4376.405	5.7		−0.013	CH	P
		11							22839.67	4377.117	4377.087	2.7		−0.030	CH	P
11									22838.96^*^	4377.253	4377.234	19.9		−0.019	CH	M
			12						22820.27^*^	4380.838	4380.852	9.6	*wN*	+ 0.014	CH—	M
	12								22819.45	4380.996	4380.990	2.7	*ut*	−0.006	CH	P
		12							22814.66	4381.915	4381.885	3.0	*u*	−0.030	CH	P
12									22814.24	4381.996	4381.989	2.5	*u*	−0.007	CH—Fe i	B
			13					6	22796.15	4385.473						A
	13							6	22795.43	4385.612	4385.611	2.5		−0.001	CH	P
13		13						12	22789.68^*^	4386.718	4386.694	5.2	*uN*	−0.024	CH	P
			14					4	22772.02	4390.120	4390.114	2.7		−0.006	CH	P
	14							4	22771.50	4390.221	4390.222	3.6	*w*	+ 0.001	CH	P
14		14						20?	22764.84^*^	4391.505	4391.488	8.6		−0.017	CH	P
			15						22747.67	4394.820	} 4394.852	11.6	*s*	$\{\begin{array}{c} +0.032\\ -0.027 \end{array}$	} Ti i	M
	15								22747.36^*^	4394.879						
15		15							22739.85^*^	4396.331	4396.309	8.2		−0.022	CH	M
			16						22723.53	4399.488	4399.482	3.6	*s*	−0.006	CH?	P
	16								22723.29	4399.535						A
16		16							22714.65^*^	4401.209						A
	17		17						22698.72^*^	4404.297	4404.277	12.0	*u*	−0.020	|Ti i—CH	M
17		17							22689.14^*^	4406.157	4406.157	5.7	*u*	0.000	CH V i	B
	18		18						22673.67^*^	4409.163	4409.128	11.6	*u*	−0.035	Fe i	M
18									22663.07^*^	4411.226	} 4411.227	5.4	*uN*	$\{\begin{array}{c} +0.001\\ -0.038 \end{array}$	CH—CH?	B
		18							22662.87	4411.265						
	19		19						22648.10^*^	4414.142	4414.124	4.3	*u*	−0.018	—CH?	B
19									22636.64	4416.377	4416.360	2.9		−0.017	—CH?	B
		19							22636.20	4416.462	4416.475	8.2	*Sd?*	+ 0.013	‖V i—Ti i?	M
	20		20						22621.65^*^	4419.303	4419.273	3.6		−0.030	Fe i p	M
20									22609.31	4421.715	4421.763	7.7	*s*	+0.048	Ti i	M
		20							22608.76	4421.823						A
	21		21						22594.30^*^	4424.653						A
21									22580.76	4427.305	4427.317	*34.6*	*S*	+ 0.012	Fe i	M
		21							22580.24	4427.407						A
	22		22						22565.75^*^	4430.250						A
22									22551.22	4433.105						A
		22							22550.46	4433.254	4433.230	*22.3*	*u*	−0.024	Fe i	M
23									22520.17	4439.217						A
		23							22519.35	4439.379	4439.358	4.3		−0.021		M
24									22487.19	4445.728						A
		24							22486.45	4445.874	4445.849	1.1	*u*	−0.025	Zr ii?	A
25									22452.10	4452.677						A
		25							22451.15	4452.865						A
26									22414.34	4460.178						A
		26							22413.37	4460.371	4460.361	6.3		−0.010	Mn i	M
27									22373.38	4468.343						A
		27							22372.25	4468.569						A

**Table 5:** CH in the Solar Spectrum A ^2^Δ—X ^2^Π (0, 1)

Laboratory											Sun					

Q_1c_	Q_1d_	Q_2c_	Q_2d_	R_1cd_	R_1dc_	R_2cd_	R_2dc_	Intensity	Wave number cm^−1^	Wavelength A	Wavelength A	Disk int. Δλ/λ	Spot	⊙—lab. A	Solar identification	Notes
					16		16	2	21150.36	4726.734						
				16		16		2	21141.13	4728.797						
							15	3	21098.50	4738.351						
					15			3	21098.30	4738.396						
				15		15		3	21090.29	4740.196						
							14	3	21047.63	4749.804						
					14			3	21047.18	4749.906						
				14		14		4	21040.28*	4751.463						
							13	3	20998.06	4761.118						
					13			3	20997.21*	4761.219						
						13		3	20991.33	4762.545						
				13				3	20991.07	4762.602						
24								2	20952.79*	4771.305						
							12	4	20948.90	4772.189						
					12			4	20948.05	4772.384						
						12		4	20943.28	4773.470						
				12				4	20942.98	4773.538						
			24					1	20931.95	4776.054						
23								2	20914.78*	4779.975						
		23						2	20912.79*	4780.429						
							11	4	20901.20	4783.081						
					11			4	20900.28	4783.292						
	23					11		4	20896.39	4784.181						
			23	11				4	20895.80	4784.317						
22								1	20878.04	4788.386						
		22						2	20876.51	4788.737						
	22		22					1	20860.86	4792.330						
							10	4	20854.80	4793.724						
					10			4	20853.60	4793.999						
						10		4	20850.71	4794.663						
				10				4	20849.84	4794.862						
21								1	20842.57	4796.536						
		21						1	20841.93	4796.684						
	21		21					1	20827.31	4800.050						
							9	4	20809.64	4804.164						
20		20			9			6	20808.15	4804.472						
						9		4	20806.18	4804.925						
				9				4	20805.12	4805.171						
	20							2	20795.31	4807.437						
			20					2	20794.70	4807.578						
19		19						3	20775.59	4812.001						
							8	5	20765.81*	4814.267						
					8			6	20764.24*	4814.630						
	19							2	20763.51	4814.800						
			19			8		7	20763.02*	4814.913						
				8				6	20761.64*	4815.233						
18		18						4	20743.87	4819.359						
	18							2	20733.14	4821.853						
			18					2	20732.67	4821.962						
							7	5	20723.41	4824.117						
					7	7		7	20721.43	4824.577						
				7				5	20719.61	4825.001						
17		17						4	20713.57	4826.409						
	17							3	20703.79	4828.687						
			17					3	20703.31	4828.800						
		16						3	20684.76	4833.131						
16								3	20684.56	4833.177						
							6	4	20682.62	4833.630						
						6		5	20681.02	4834.004						
					6			6	20680.25	4834.185						
				6				5	20678.88	4834.505						
	16		16					5	20675.54*	4835.285						
		15						4	20657.22	4839.573						
15								4	20656.75	4839.684						
	15		15					5	20648.92	4841.520						
							5	5	20643.44	4842.804						
						5		5	20642.36	4843.058						
					5			5	20640.66	4843.457						
				5				5	20639.68	4843.686						
		14						5	20631.09	4845.704						
14								6	20630.61*	4845.817						
	14		14					6	20623.71	4847.438						
		13						6	20606.45*	4851.499						
13							4	7	20605.89	4851.630						
						4		6	20605.36*	4851.755						
					4			5	20602.47	4852.435						
				4				5	20601.78	4852.598						
	13		13					6	20599.91	4853.038						
		12						5	20583.50	4856.908						
12								6	20582.64*	4857.110						
			12					6	20577.87	4858.236						
	12							6	20577.42	4858.343						
							3	4	20570.66	4859.939						
						3		4	20570.13	4860.065						
							‡	1	20566.39	4860.949						
					3			6	20565.74	4861.103						
				3				6	20565.43	4861.176						
		11						5	20562.10	4861.963						
11								6	20561.04	4862.213						
			11					6	20557.26	4863.107						
	11							6	20556.64	4863.255						
		10						6	20542.32	4866.644						
10								6	20541.10	4866.932						
			10					6	20538.26	4867.606						
	10					2	2	8	20537.45	4867.799	4867.874	11.5	*u*	+ 0.075	Co i	M
							‡	2	20531.58	4869.190						
				2	2			7	20530.44	4869.460	4869.469	4.1	*u*	+ 0.009	Fe i	M
		9						6	20524.30	4870.916	4870.94	0.8		+ 0.02	Ni i	M
9								6	20522.84	4871.263						A
			9					6	20520.91	4871.721	4871.683	3.1		−0.038		A
	9							6	20519.81	4871.982	4871.935	9.2	*u*	−0.047	Fe i p	M
						1	1	5	20515.74	4872.950						A
		8						6	20508.01	4874.786	4874.793	4.3	*w?*	+ 0.007	Ni i	M
8								6	20506.31	4875.190	4875.198	0.6		+ 0.008	CH?	P
			8					6	20505.26	4875.439	4875.492	*8.4*	*S*	+ 0.053	V i	M
	8							6	20503.87	4875.771	4875.739	0.7		−0.032	Fe i p	M
							‡	3	20497.98	4877.172						
				1	1			7	20496.23*	4877.589	4877.590	4.3	*u*	+ 0.001	Fe i	M
		7						6	20493.48	4878.243	4878.225	*24.2*	*w*	−0.018	Fe i	M
7			7					9	20491.43	4878.730	4878.721	0.6		−0.009	CH	P
	7							G	20489.56	4879.175	4879.150	0.4		−0.025	CH?	P
		6						5	20480.80*	4881.262	4881.267	0.4		+ 0.005	CH?	P
			6					6	20479.27*	4881.627						A
6								G	20478.34*	4881.849						A
	6							7	20476.93*	4882.185	4882.148	*13.9*	*u*	−0.037	Fe i	M
		5						5	20470.05	4883.826						A
			5					5	20468.83	4884.117						A
							‡	1	20468.24	4884.258						
5								6	20467.00	4884.555	4884.598	4.3	*o*	+ 0.043	Cr ii	M
	5							7	20465.98	4884.798	4884.803	1.5		+ 0.005	CH?—	B
		4						5	20461.26	4885.924	4885.950	2.4	*s*	+ 0.026	Cr i	M
			4				‡	6	20460.46	4886.116	4886.086	0.2		−0.030	CH?	P
							‡	1	20459.83	4886.266						
							‡	1	20458.12	4886.674						
4							‡	7	20457.36*	4886.856	4886.845	0.6	*s*	−0.011	V i—CH?	B
	4							6	20456.67*	4887.021	4887.009	12.9	*s*	−0.012	‖Cr i Ni i	M
		3						4	20454.60	4887.514	4887.533	1.0		+ 0.019		M
			3					4	20454.11	4887.631						A
							‡	1	20453.37	4887.808						
		2	2				‡	3	20450.57	4888.477						
				3				6	20449.34	4888.768						A
					3		‡	6	20449.01	4888.850	4888.829	1.5		−0.021	—CH?	B
							‡	1	20444.50	4889.930						
				2	2			4	20442.89*	4890.315						
			3					2	20367.91	4908.318						
		3						2	20367.11	4908.510						
							‡	1	20366.17	4908.737						
							‡	1	20365.41	4908.919						
	3							2	20361.98	4909.746						
3								2	20361.46	4909.871						
			4					1	20345.30	4913.773						
		4						1	20344.31	4914.011						
							‡	1	20343.19	4914.283						
	4							2	20340.94	4914.827						
4								2	20340.23	4914.997						
			5					2	20325.13	4918.650						
		5						2	20323.98	4918.927						
							‡	2	20323.26	4919.101						
	5							3	20321.82	4919.451						
5								3	20320.85	4919.686						
			6					2	20307.32	4922.963						
		6						2	20305.76	4923.342						
	6							2	20304.63	4923.614						
6								3	20303.26	4923.948						
	22							1	20301.04	4924.487						
			22					1	20299.58	4924.839						
			7					4	20291.61	4926.775						
	7	7						5	20289.45	4927.299						
7								4	20287.51	4927.770						
	21							2	20285.93	4928.154						
22								2	20285.35	4928.294						
	22		21					2	20284.55	4928.489						
			8					4	20277.97	4930.088						
	8							4	20276.17	4930.526						
		8						4	20275.20	4930.762						
8	20		20					5	20273.67	4931.134						
21								1	20272.33	4931.460						
		21						1	20271.16	4931.744						
			9					4	20266.41	4932.901						
	9							4	20264.88	4933.273						
	19	9	19					5	20263.12	4933.702						
9								4	20261.84	4934.013						
20								2	20260.69	4934.294						
		20						2	20260.04	4934.453						
			10					3	20256.94	4935.206						
	10							3	20255.70	4935.509						
	18		18					3	20254.61	4935.773						
		10						3	20252.96	4936.176						
10								4	20251.99	4936.412						
19								3	20251.35	4936.569						
		19						3	20250.77	4936.710						
			11					3	20249.64	4936.986						
	11							4	20248.49	4937.265						
	17		17					3	20248.07	4937.369						
		11						3	20244.80	4938.166						
11, 18			12					4	20244.16	4938.321						
	12	18	16					5	20243.38	4938.511						
	16							3	20242.97	4938.613						
			13					3	20241.03	4939.085						
			15					3	20240.55	4939.202						
	13, 15							4	20240.22	4939.282						
			14					3	20239.85	4939.373						
	14							3	20239.26	4939.518						
		12						3	20238.70	4939.654						
12, 17		17						4	20238.13	4939.792						
		13						3	20234.42	4940.700						
13, 16		16						5	20234.21	4940.750						
14, 15		14, 15						5	20232.34	4941.208						

* Blend.

‡ Satellite lines as follows:

**Table:** 

Designation	*J*	Laboratory	
		Wave No.	Wavelength

R_Q 2c 1d_; R_Q2d 1c_	3; 3	20566.39	4860.949
R_Q 2c 1d_; R_Q2d 1c_	2; 2	20531.58	4869.190
R_Q 2c 1d_; R_Q2d 1c_	1; 1	20497.98	4877.172
Q_R 1 2d_	5	20468.24	4884.258
Q_R 1 2d_	4	20460.46	4886.116
Q_R 1 2d_	4	20459.83	4886.266
Q_P 1 2d_	4	20458.12	4886.674
Q_P 1 2d_	4	20457.36	4886.856
Q_R 1 2c_; Q_R 1 2d_	3; 3	20453.37	4887.808
Q_P 2 1c_; Q_P 2 1d_	3; 3	20450.57	4888.477
Q_R 1 2c_; Q_R 1 2d_	2; 2	20449.01	4888.850
Q_P 2 1c_; Q_P 2 1d_	2; 2	20444.50	4889.930
P_Q 1d 2c_	3	20366.17	4908.737
P_Q 1c 2d_	3	20365.41	4908.919
P_Q 1c 2d_	4	20343.19	4914.283
P_Q 1c 2d_	5	20323.26	4919.101

**Table 6:** CH in the Solar Spectrum A ^2^Δ—X ^2^Π (1,2)

Laboratory														
P_1cd_	P_1dc_	P_2cd_	P_2dc_	Q_1c_	Q_1d_	Q_2c_	Q_2d_	R_1cd_	R_1dc_	R_2cd_	R_2dc_	Intensity	Wave number cm^−1^	Wavelength A
									13		13	1	21083.78	4741.660
								13		13		2	21077.49	4743.076
									12		12	4	21040.28*	4751.463
								12		12		1	21034.82	4752.697
											11	2	20998.22	4760.982
									11			3	20997.21*	4761.219
										11		1	20993.57	4762.035
								11				1	20992.87	4762.194
											10	2	20956.62	4770.432
									10			2	20955.52	4770.682
										10		2	20952.79*	4771.305
								10				2	20951.91	4771.505
											9	2	20916.02	4779.692
									9			2	20914.78*	4779.975
										9		2	20912.79*	4780.429
								9				2	20911.85	4780.644
											8	2	20876.26	4788.794
									8			2	20874.73	4789.145
										8		2	20873.82	4789.354
								8				2	20872.56	4789.644
											7	2	20837.84	4797.625
									7	7		3	29835.83	4798.089
								7				2	20834.15	4798.475
											6	2	20800.39	4806.263
										6		2	20798.97	4806.591
									6			2	20798.14	4806.784
				17		17		6				3	20796.78	4807.097
					17		17					1	20787.35	4809.279
				16		16						2	20774.31	4812.296
					16		16					5	20765.81*	4814.267
											5	6	20764.24*	4814.630
										5		7	20763.02*	4814.913
									5			6	20761.64*	4815.233
								5				2	20760.55	4815.486
						15						2	20752.52	4817.350
				15								1	20751.93	4817.486
					15		15					2	20744.41	4819.232
						14						2	20731.59	4822.214
				14								2	20730.92	4822.370
											4	2	20729.75	4822.640
										4		2	20728.91	4822.837
									4			3	20726.33	4823.438
								4				3	20725.64	4823.597
					14		14					3	20724.38	4823.891
						13						2	20711.85	4826.844
				13								2	20710.99	4827.009
					13		13					2	20705.27	4828.342
											3	2	20696.75	4830.332
										3		2	20695.97	4830.513
						12					‡	2	20692.96	4831.216
				12					3			4	20692.14	4831.408
								3				3	20691.71	4831.508
							12					2	20687.60	4832.467
					12							2	20686.99	4832.610
						11						5	20675.54*	4835.285
				11								2	20674.45	4835.541
							11					2	20670.88	4836.376
					11							2	20670.20	4836.535
										2	2	2	20665.81	4837.562
											‡			4838.899
						10		2	2			5	20659.09	4839.135
				10								3	20658.03	4839.384
							10					3	20655.35	4840.012
					10							3	20654.55	4840.200
						9						3	20644.23	4842.620
				9								3	20642.80	4842.954
							9					3	20641.02	4843.371
					9					1	1	5	20639.87	4843.641
						8						6	20630.61*	4845.817
				8								3	20629.01	4846.191
											‡	2	20628.34	4846.350
							8					3	20627.96	4846.440
					8			1	1			5	20626.65	4846.746
						7						3	20618.58	4848.644
				7			7					4	20616.61	4849.108
					7							3	20614.78	4849.537
						6						3	20607.97	4851.141
							6					6	20606.45*	4851.499
				6								6	20605.36*	4851.755
					6							3	20604.23	4852.020
						5						3	20598.95	4853.265
							5					3	20597.80	4853.537
				5								3	20596.06	4853.945
					5							3	20595.01	4854.194
						4						3	20591.61	4854.995
							4					3	20590.65	4855.221
				4								3	20587.97	4855.854
					4							3	20587.27	4856.019
						3						2	20585.87	4856.348
							3					2	20584.67	4856.631
						2	2					6	20582.64*	4857.110
				3								3	20581.17	4857.459
					3							3	20580.69	4857.572
				2	2							2	20575.52	4858.791
			3									2	20502.61	4876.070
		3									‡	2	20501.28	4876.387
											‡	2	20500.65	4876.535
	3											1	20497.27	4877.340
3												7	20496.23*	4877.589
			4									5	20480.80*	4881.262
		4										6	20479.27*	4881.627
											‡	6	20478.34*	4881.849
	4											7	20476.93*	4882.185
4												1	20475.50	4882.526
			5									2	20460.87	4886.017
		5										1	20459.59	4886.322
											‡	1	20458.64	4886.549
	5											7	20457.36*	4886.856
5												6	20456.67*	4887.021
			6									4	20442.89*	4890.315
		6										2	20441.13	4890.735
	6											2	20439.97	4891.014
6												2	20438.76	4891.302
			7									2	20426.06	4894.343
	7	7										3	20424.13	4894.807
7												2	20422.38	4895.227
			8									2	20411.40	4897.859
	8											2	20409.71	4898.265
		8										2	20408.61	4898.528
8												2	20407.41	4898.816
			9									2	20398.60	4900.933
	9											2	20397.08	4901.296
		9										2	20395.39	4901.704
9												2	20394.16	4901.999
			10									2	20387.46	4903.610
	10											2	20386.24	4903.905
		10										2	20383.50	4904.563
10												2	20382.60	4904.779
			11									2	20377.89	4905.913
	11											2	20376.83	4906.169
		11										2	20373.31	4907.017
11												2	20372.53	4907.205
			12									2	20370.00	4907.814
	12											2	20368.99	4908.057
		12										2	20364.85	4909.055
12			13									2	20364.14	4909.227
	13											2	20363.15	4909.463
		14										2	20359.49	4910.347
	14											2	20358.74	4910.529
		13										2	20357.79	4910.756
13												2	20357.56	4910.812
			15									2	20356.53	4911.062
	15											2	20356.01	4911.186
	17		17									2	20355.21	4911.379
			16									2	20354.79	4911.481
	16											2	20354.36	4911.583
14		14										3	20352.25	4912.094
15		15										2	20348.75	4912.939
16, 17	16	17										1	20346.28	4913.535

* Blend.

‡ Satellite lines as follows :

**Table:** 

Designation	*J*	Laboratory	
		Wave number	Wavelength

R_Q 2d 1c_	3	20692.96	4831.216
R_Q 2c 1d_ ; R_Q 2d 1c_	2; 2	20660.10	4838.899
R_Q 2c 1d_ ; R_Q 2d 1c_	1; 1	20628.34	4846.350
P_Q 1d 2c_	3	20501.28	4876.387
P_Q 1c 2d_	3	20500.65	4876.535
P_Q 1c 2d_	4	20478.34	4881.849
P_Q 1c 2d_	5	20458.64	4886.549

**Table 7:** CH in the Solar Spectrum B ^2^Σ^−^—X ^2^Π (0, 0)

Laboratory									Sun					

P_1_	P_2_	Q_1_	Q_2_	R_1_	R_2_	Intensity	Wave number cm^−1^	Wavelength A	Wavelength A	Disk int. Δλ/λ	Spot	⊙—lab. A	Solar identification	Notes
					7	25	25823.37	3871.363	3871.392	226		+0.029	CN (CH)	M
					6	27	25822.18	3871.542	3871.563	24.5		+0.021	CH CN—Ni i	B
				7		34	25821.35	3871.666	3871.651	15.7		−0.015	La ii CH	B
					8	33	25820.64	3871.773	3871.758	41.8		−0.015	Fe i (CH)	M
				6		30	25819.84	3871.893	3871.903	14.4		+0.010	CH	P B
				8		30	25818.73	3872.059	3872.062	25.8		+0.003	CN CH	B
					5	20	25817.23	3872.284	3872.273	20.9		−0.011	|CN—CH	B
				5		27	25814.51	3872.692	3872.732	31.8		+0.040	|CN V i? CH?	B
					9	26	25813.61	3872.827	3872.834	21.9		+0.007	CH	P
				9		27	25811.97	3873.073	3873.090	30		+0.017	CH—	B
					4	16	25808.78	3873.552	3873.575	20.9		+0.023	CN CH?	B
				4		21	25805.46	3874.050	3874.060	47.6		+0.010	Fe i (CH)	M
					10	24	25802.18	3874.543	3874.524	23.4		−0.019	—CH	B
				10		26	25800.65	3874.773	3874.776	22.4		+0.003	CH CN	B
					3	13	25797.09	3875.308	3875.290	24.5		−0.018	Ti i—CN	M
				3		17	25792.77	3875.956	3875.948	19.3		−0.008	CN—CH	B
					11	24	25785.97	3876.978	3876.978	26.9		0.000	CN—CN (CH)	M
				11		25	25784.56	3877.191	3877.198	19.1		+0.007	CH	P
					2	8	25782.80	3877.455	3877.451	19.1		−0.004	CN CH	B
				2		12	25776.44	3878.412	3878.412	33.2		0.000	CH CN—CN	B
					1	4	25774.08	3878.768	3878.747	61.9		−0.021	Fe i|V ii CN	M
					12	22	25764.70	3880.180	3880.190	25.7		+0.010	CH	P
				12		23	25763.37	3880.380	3880.394	22		+0.014	CN (CH)	M
				1		8	25756.06	3881.482	3881.490	3.2		+0.008	CH	P
					13	20	25737.87	3884.225	3884.222	10.8		−0.003	CH	P P
				13		20	25736.60	3884.416	3884.440	9.5		+0.024	CH	P
			1			10	25723.40	3886.410	3886.428	31.6		+0.018	CH—	B
			2			20	25706.87	3888.909	3888.938	20.8		+0.029	CH	P
		1			14 }	27	$\{\begin{array}{c} 25705.52\\ 25705.08 \end{array}$	3889.113	3889.105	39.4		−0.008	CH	P
								3889.180	3889.231	14.7		+0.051	CH—	B P
				14		18	25703.82	3889.370	3889.358	22.1		−0.012	CH Fe i	B
		2				31	25700.66	3889.848	3889.848	22.1		0.000	CH	P
	1					5	25698.25	3890.213	3890.196	16.4		−0.017	V i—Mg i?	M
			3			29	25695.99	3890.555	3890.568	16.7		+0.013	‖CH—Nd ii	B
		3				39	25691.81	3891.188	3891.198	*20.4*		+0.010	CH	P
			4			38	25682.60	3892.584	3892.591	*16.4*		+0.007	CH	P
1						20?	25680.35	3892.925	3892.898	26.2		−0.027	|Fe i V i (CH)	M
		4				47	25679.40	3893.069	3893.074	30.3		+0.005	CH	P
			5		15 }	49	$\{\begin{array}{c} 25666.16\\ 25665.63 \end{array}$	3895.077	3895.088	21.2		+0.011	CH—Ce ii	B
								3895.157	3895.167	12.6		+0.010	CH	P
				15		20?	25664.45	3895.337	3895.334	19.3	*u*	−0.003	CH—	B
		5				54	25663.66	3895.457	3895.448	42.3	*u*	−0.009	Fe i p—CH|	B
	2					13	25656.61	3896.527	3896.534	5.9		+0.007	Zr i? CH?	B
2						18	25650.29	3897.487	3897.458	*28.4*	*u*	−0.029	Fe i (CH)	M
			6			51	25646.44	3898.072	3898.094	18.7	s*	+0.022	CH	P
		6				58	25644.34	3898.391	3898.394	22.4	*u*	+0.003	CH	P
			7			55	25623.25	3901.600	3901.598	*20*	*u*	−0.002	CH	P
		7				65	25621.54	3901.860	3901.866	21.4	*u*	+0.006	CH	P
	3					25?	25620.87	3901.962	3901.977	13.3	*u*	+0.015	CH—Cr i	B
					16	6	25618.96	3902.253	3902.262	*21.5*	*s*	+0.009	V i (CH)	M
				16		7	25617.86	3902.421	3902.430	14.9	*u*	+0.009	Gd ii—CH	B
3						23	25616.58	3902.616	3902.632	15.9		+0.016	CH	P
			8			59	25596.57	3905.667	3905.679	46.1		+0.012	|CH Fe i p	B
		8				64	25595.02	3905.904	3905.905	25.9	*u*	+0.001	CH	P
	4					23	25582.80	3907.770	3907.774	12.8	*u*	+0.004	Cr i CH	B
4						27	25579.53	3908.268	3908.274	13.8		+0.006	CH	P
			9			59	25566.07	3910.327	3910.334	24.3		+0.007	CH	P
		9				64	25564.66	3910.542	3910.534	30.4		−0.008	CH	P
					17	M	25563.94	3910.653	3910.667	16	*u*	+0.014		M
				17		1	25562.81	3910.825	3910.849	26.1	*u*	+0.024	Fe i	M
	5					28	25542.01	3914.010	3914.013	*16.6*		+0.003	CH	P
5						32	25539.32	3914.422	3914.426	26.1	*u*	+0.004	CH	P
			10			59	25531.63	3915.601	3915.612	23		+0.011	CH	P
		10				63	25530.36	3915.796	3915.811	32	*s*?	+0.015	CH—Cr i	B
	6			18		30	25498.30	3920.720	3920.729	27.8	*u*	+0.009	CH—	B
6						33	25496.00	3921.073	3921.049	42.1	*s*	−0.024	Cr i (CH)	M
			11			57	25492.92	3921.547	3921.556	27.8	*w*	+0.009	CH	P
		11				59	25491.85	3921.712	3921.716	26.8	*u*	+0.004	CH	P
	7					32	25451.42	3927.941	3927.933	159	*S*	−0.008	Fe i (CH)	M
7			12			86	25449.63*	3928.218	3928.217	49.4	*u*	−0.001	CH	P
		12				63	25448.80	3928.346	3928.345	32.8		−0.001	CH	P
	8		13			80	25401.73*	3935.625	3935.645	31	*uN*	+0.020	CH	P
		13				65?	25401.03	3935.734	3935.73	18		0.00	CH	P
8						38	25399.72	3935.937	3935.979	59.4	*s*	+0.042	Co i (CH)	M
			14			65?	25348.77	3943.848	3943.820	39.6	*uN*	−0.028	CH	P
	9	14				78	25348.16*	3943.943	3943.918	8.9		−0.025	Ce ii—CH|	B
9						37	25346.65	3944.178	3944.179	33.1	*uN*	+0.001	|CH—	B
	10					34	25291.71	3952.746	3952.756	6.3		+0.010	CH	P
10			15			79	25290.26*	3952.973	3952.982	28	s*	+0.009	CH	P
		15				50?	25289.46	3953.098	3953.084	12.5	*o*?	−0.014	CH	P
	11					33	25231.54	3962.172	3962.184	21.4	*u*	+0.012	CH—	B
11						35.5	25230.12	3962.395	3962.398	8.8		+0.003	CH	P
			16			} 36	$\{\begin{array}{c} 25225.46\\ 25224.93 \end{array}$	3963.127	3963.115	*44.9*	*u*	−0.012	Fe i (CH)	M
		16						3963.211	3963.233	28.5	*u*	+0.022	CH	P
	12					31	25167.58	3972.242	3972.267	15.8	*w?*	+0.025	CH	P
12						32	25166.26	3972.450	3972.44	13.3	*uN*	−0.01	Co i CH	B
			17			} 18	$\{\begin{array}{c} 25153.89\\ 25153.48 \end{array}$	3974.404	3974.396	27.3	*u*	−0.008	Fe i (CH)	M
		17						3974.469	3974.497	25.9	*u*	+0.028	CH—	B
	13					27.5	25099.63	3982.996	3983.008	15.3		+0.012	CH—	B
13						30	25098.41	3983.190	3983.199	16.5	*u*	+0.009	CH—	B
			18			} 4	$\{\begin{array}{c} 25074.46\\ 25074.17 \end{array}$	3986.994	}3986.994	13.7	*uN*	$\{\begin{array}{c} 0.000\\ -0.046 \end{array}$	CH—	B
		18						3987.040						M
	14					25	25027.29	3994.509	3994.514	17.6	*u*	+ 0.005	CH—Co i|	B
14						26	25026.13	3994.693	3994.683	16.5	*u*	−0.010	Ti i Nd ii CH	B
		19					24986.23	4001.074	4001.110	6.0		+ 0.036		A
	15					20	24950.24	4006.845	4006.825	10.5	*uN*	−0.020	—CH	B
15						21	24949.16	4007.018	4006.997	14.5	*u*	−0.021	CH	P
	16					17.5	24868.31	4020.046	4020.029	8.5	*u?*	−0.017	—CH	B
16						18	24867.27	4020.214	4020.193	10.7	*u*	−0.021	—CH	B
	17					13	24780.76	4034.249	4034.232	12.9	*u*	−0.017	—CH	B
17						13	24779.74	4034.415	4034.386	9.9		−0.029	CH	P
	18					6.5	24686.90	4049.587	4049.565	10.1	*u*	−0.022	CH	P
18						5.5	24685.91	4049.750	4049.731	11.6	*u*	−0.019	CH	P
	19						24585.82	4066.237	4066.220	6.4	*u*	−0.017	—CH	B
19							24584.86	4066.396	4066.373	18.7	*s*	−0.023	Co i	M
20							24476.39	4084.417						A

* Blend.

‡ Satellite line as follows:

**Table:** 

Designation	*J*	Laboratory	
		Wave number	Wavelength
P_Q 12_	1	25698.25	3890.213

**Table 8:** CH in the Solar Spectrum B ^2^Σ^−^—X ^2^Π (1, 0)

Laboratory									Sun				

P_1_	P_2_	Q_1_	Q_2_	R_1_	R_2_	Intensity	Wave number cm^−1^	Wavelength A	Wavelength A	Disk int. Δλ/λ	⊙—lab. A	Solar identification	Notes
					3	0.9	27561.60	3627.202	3627.169	5	−0.033		B
					2, 1	1.0	27559.78	3627.442	3627.456	3.2	+0.014	CH	P
					4	} 1.9	$\{\begin{array}{c} 27558.31\\ 27557.74 \end{array}$	3627.635	3627.623	11.3	−0.012	Mg i? CH	B
				3				3627.710	3627.715	0.7	+0.005	V ii Ti ii	M
				4		1.5	27554.95	3628.078	3628.098	21.8	+0.020	Fe i	M
				2		0.9	27553.45	3628.275	3628.279	6.8	+0.004	CH	P
					5	1.4	27548.01	3628.992	3629.006	4.4	+0.014	CH	P
				5		1.8	27545.17	3629.366	3629.352	4.4	−0.014	CH	P
				1		0.7	27542.25	3629.751	3629.737	14	−0.014	Mn i	M
					6	1.2	27530.49	3631.301	3631.265	45.4	−0.036		A
				6		1.5	27528.25	3631.596	3631.586	31.2	−0.010		M
			1			1.0	27515.40	3633.292	3633.308	6.6	+0.016	CH	P
					7	1.1	27505.35	3634.620	3634.618	5	−0.002	CH	P
				7		1.0	27503.40	3634.878	3634.865	5.1	−0.013	CH	P
		1				1.5	27497.52	3635.655	3635.652	4.7	−0.003	Ti i p? CH	B
	1		2		‡	2.1	27492.88*	3636.269	3636.238	16.5	−0.031	Fe i	M
		2				2.3	27486.64	3637.094	3637.058	10.7	−0.036	Fe i	M
1						1.1	27475.26	3638.601	3638.605	2	+0.004	CH	P
			3			2.4	27472.90	3638.913	3638.905	4.4	−0.008	CH	P
					8	M	27472.28	3638.996	3639.030	9.1	+0.034	V i—	M
				8		M	27470.15	3639.277	3639.285	14.3	+0.008		M
		3				2.9	27468.73	3639.466	3639.450	18.7	−0.016	Co i	M
	2					M	27448.48	3642.151	3642.147	22.8	−0.004	—CH	B
			4			2.9	27447.38	3642.297	3642.281	5.9	−0.016	CH	P
		4				3.6	27444.25	3642.712	3642.682	31.8	−0.030	Ti i	M
2						1.5	27442.25	3642.978	3642.971	5.4	−0.007	CH	P
					9	0.1?	27429.39	3644.686	3644.695	15.8	+0.009	Cr ii	M
				9		0.1?	27428.09	3644.859					A
			5			3.2	27415.56	3646.525	3646.504	6.6	−0.021	CH	P
		5				3.9	27413.08	3646.855	3646.837	9.9	−0.018	—CH	B
	3					1.3	27406.76	3647.696	3647.669	59.6	−0.027	Co i	M
3						1.8	27402.50	3648.263	3648.228	14	−0.035	Fe i p—CH	B
			6			3.6	27377.09	3651.649	3651.654	28	+0.005	Ni i Cr ii	M
		6				4.0	27375.02	3651.925	3651.921	25.6	−0.004	—CH	B
	4					1.8	27359.76	3653.962	3653.979	20.2	+0.017	Fe i	M
4						2.1	27356.49	3654.398	3654.386	5.1	−0.012	CH	P
			7			3.5	27331.61	3657.725	3657.711	21.2	−0.014	Ni i Fe i|	M
		7				3.6	27329.83	3657.963					A
	5					2.1	27306.82	3661.046	3661.038	10.4	−0.008	CH	P
5						2.3	27304.15	3661.404	3661.372	22.6	−0.032	Sm ii Fe i—V ii	M
			8			2.2	27278.56	3664.839	3664.828	13	−0.011	CH	P
		8				2.3	27277.08	3665.038	3665.028	13.9	−0.010	—CH	B
	6					2.2	27247.74	3668.984	3668.969	23	−0.015	Ti i	M
6						2.6	27245.47	3669.290	3669.244	35	−0.046	Ni i	M
			9			1.0	27217.46	3673.066	3673.087	32.7	+0.021	Fe i—	M
		9				1.0	27216.18	3673.239	3673.226	17.2	−0.013	—CH	B
	7					2.4	27182.16	3677.836	3677.855	38.5	+0.019	Cr ii	M
7						2.6	27180.21	3678.100	3678.100	12.9	0.000	CH	P
	8					2.2	27109.90	3687.640	3687.660	41.7	+0.020	Fe i	M
8						2.5	27108.14	3687.878	3687.866	7.6	−0.012	CH	P
	9					1.2	27030.43	3698.482	3698.477	7.3	−0.005	—CH	B
9						1.2	27028.86	3698.697	3698.694	6.8	−0.003	CH	P
	10					0.5	26942.95	3710.490					A
10						0.5	26941.64	3710.670	3710.638	10.8	−0.032		

* Blend.

‡ Satellite line as follows:

**Table:** 

Designation	*J*	Laboratory	
		Wave No.	Wavelength
P_Q 1 2_	1	27492.88	3636.269

**Table 9:** CH in the Solar Spectrum B ^2^Σ^−^— X ^2^Π (1, 1)

Laboratory									Sun					

P_1_	P_2_	Q_1_	Q_2_	R_1_	R_2_	Intensity	Wave number cm^−1^	Wavelength A	Wavelength A	Disk int. Δλ/λ	Spot	⊙—lab. A	Solar identification	Notes
					4	1	24835.75	4025.316	4025.308	3.5		−0.008	CH	P
					3	2	24835.22	4025.402	4025.429	11.9	*u*	+0.027	|Ni i Cr i	M
				4		2	24832.45	4025.851	4025.823	15.9	*u*	−0.028		M
				3	5	3.5	24830.76*	4026.125	4026.168	10.7	*sd*	+0.043	Cr i	M
					2	0.5	24829.60	4026.313	4026.310	1.7		−0.003	CH	P
				5	1	2.5	24828.06*	4026.562	4026.542	10.4	*s*	−0.020	Ti i	M
				2		1	24823.62	4027.283	4027.251	2.4	*s*	−0.032	Zr i—CH	B
					6	0.5	24819.66	4027.926	4027.943	7.0	*u*	+0.017	CH—	B
				6		0.5	24817.25	4028.317	4028.346	22.3	*u*	+0.029	Ti ii	M
				1		0.5	24810.17	4029.466	4029.443	2.5	*u*	−0.023	CH	P
					7	0.5	24801.91	4030.808	4030.763	53.1	*S*	−0.045	Mn i	M
				7		0.5	24799.95	4031.127	4031.116	4.2	*u*	−0.011	Cr i	M
			1			0.5	24783.46	4033.809	4033.790	2.4	*u*	−0.019	CH	P
		1				2	24765.72	4036.699	4036.670	1.5		−0.029	CH	P
			2			2	24762.79	4037.177	4037.121	10.9	*u*	−0.056		M
	1				‡	0.5	24761.26	4037.426	4037.438	1.2		+0.012	CH	P
		2				3.5	24756.88	4038.140	4038.124	3.2	*s*	−0.016		M
			3			3.5	24746.14	4039.893	4039.864	2.2	*s*	−0.029		M
1						1.5	24743.41	4040.339	4040.310	4.0	*s*	—.0.029	Ti i—CH	B
		3				4	24742.16	4040.543	4040.514	6.9	*u*	−0.029	—CH	B
			4			4.5	24724.98	4043.350	4043.346	5.9	*uN*	−0.004	CH	P
		4				4.5	24721.89	4043.856	4043.906	22.0	*u*	+0.050	Fe i	M
	2					0.5	24718.50	4044.410	4044.380	1.7		−0.030	CH	P
2						1	24712.51	4045.391	4045.390	25.0	*u*	−0.001	Co i	M
			5			4	24698.42	4047.699	4047.673	5.4	*s*	−0.026	Y i—CH	B
		5				5	24696.01	4048.093	4048.072	5.9	*u*	−0.021	—CH	B
	3					1	24680.01	4050.718	4050.680	14.1	*u*	−0.038	Fe i	M
3						2	24675.90	4051.392						A
			6			4	24666.30	4052.969	4052.940	7.9	*u*	−0.029	Ti i—CH	B
		6				5	24664.35	4053.290	4053.271	15.0	*u*	−0.019	Fe i	M
	4					2	24637.26	4057.747	4057.733	3.2		−0.014	CH	P
4						3	24634.33	4058.229	4058.221	24.9	*s*	−0.008	Co i Fe i‖	M
			7			3.5	24628.23	4059.234	4059.222	5.7	*u*	−0.012	CH	P
		7				4	24626.54	4059.513	4059.502	5.7	*u*	−0.011	CH	P
	5					4	24589.48	4065.632	4065.587	3.7	*u*	−0.045	Ti i CH	B
5						3.5	24587.06	4066.032	4066.004	3.0	*u*	−0.028	Fe i p—CH	B
			8			2	24583.70	4066.588	4066.590	19.7	*u*	+0.002	Fe i	M
		8				3	24582.18	4066.839	4066.820	7.6	*u*	−0.019	CH	P
	6					2.5	24536.85	4074.352	4074.332	4.4	*sN*	−0.020	Ti i W i	M
6						3	24534.69	4074.711	4074.684	14.0	*u*	−0.027	Fe i p	M
			9			1.5	24532.00	4075.158	4075.103	15.2	*u*	−0.055		M
		9				0.5	24530.85	4075.349	4075.316	7.6	*uN*	−0.033	—CH	B
	7					3	24478.67	4084.036	4083.999	4.9	*u*	−0.037	CH?	P
7						3.5	24476.75	4084.357	4084.327	6.4	*u*	−0.030	CH	P
	8					2.5	24414.86	4094.710	4094.698	5.9	*u*	−0.012	CH	P
8						2.5	24413.13	4095.000	4094.938	25.4	*s*	−0.062	Ca i	M
	9					1	24344.81	4106.493	4106.432	19.5	*u*	−0.061	Fe i	M
9						1	24343.31	4106.746	4106.730	3.8	*u*	−0.016	CH	P
	10					0.3	24268.04	4119.483	4119.526	7.1	*s*	+0.043	Fe ii p	M
10						0.3	24266.88	4119.680	4119.671	5.6	*sd*?	−0.009	Fe i p—CH	B

* Blend.

‡ Satellite line as follows:

**Table:** 

Designation	*J*	Laboratory	
		Wave No.	Wavelength
P_Q 1 2_	1	24761.26	4037.426

**Table 10:** CH in the Solar Spectrum C ^2^Σ^+^—X ^2^Π (0, 0)

Laboratory									Sun				

P_1_	P_2_	Q_1_	Q_2_	R_1_	R_2_	Intensity	Wave number cm^−1^	Wavelength A	Wavelength A	Disk int. Rowl. est.	⊙—lab. A	Solar identification	Notes
				26		0.2	32388.47	3086.622	3086.636	[−1]	+0.014		M
					26	0.2	32387.24	3086.739	3086.787	[ 4]	+0.048	Co i	A
				25		0.25	32384.23	3087.027	3086.988	[−2]	−0.039		M
					25	0.25	32383.11	3087.133					A
				24		0.35	32377.11	3087.705	3087.693	[−1]	−0.012		M
					24	0.35	32376.03	3087.809	3087.843	[ 0Nd?]	+0.034	Co i—Cr ii?	M
				23		0.5	32367.58	3088.615	3088.610	[−1]	−0.005		M
					23	0.5	32366.42	3088.725	3088.752	[ 2]	+0.027		M
				22		1	32355.73	3089.746	3089.745	[ 2]	−0.001	OH	M
					22	1	32354.53	3089.860	3089.868	[ 2]	+0.008	OH	M
				21		1.5	32341.55	3091.100	3091.071	[ 4]	−0.029	Mg i	M
					21	2	32340.55	3091.196	3091.213	[ 1N]	+0.017	CH OH‖	B
				20		2	32325.75	3092.612	3092.598	[−3]	−0.014	CH OH	B
					20	2.5	32324.78	3092.704	3092.712	[ 4]	+0.008	Al i	M
				19		2.5	32308.24	3094.288	3094.295	[ 1]	+0.007	CH—	B
					19	3.5	32307.55	3094.354	3094.364	[−1]	+0.010	CH	P
				18		} 5	$\{\begin{array}{c} 32289.14\\ 32288.60 \end{array}$	3096.119	}3096.138	[ 2]	$\{\begin{array}{c} +0.019\\ -0.031 \end{array}$	} OH Cr ii	M
					18			3096.169					
				17		} 6	$\{\begin{array}{c} 32268.92\\ 32268.35 \end{array}$	3098.058	3098.072	[ 0]	+0.014	CH	P
					17			3098.113					A
				16		} 8	$\{\begin{array}{c} 32247.47\\ 32247.15 \end{array}$	3100.119	}3100.150	[−1N]	$\{\begin{array}{c} +0.031\\ +0.001 \end{array}$	} CH CH	
					16			3100.149					
				15	15	10	32224.58	3102.321	3102.299	[ 3]	−0.022	CH—V ii	B
				14	14	11	32201.35	3104.559	3104.571	[ 2N]	+0.012	CH—Ti ii?	B
				13	13	13	32177.15	3106.894	3106.907	[ 1]	+0.013	CH	P
				12	12	14	32152.07	3109.318	3109.333	[ 3]	+0.015	OH CH	B
				11	11	14.5	32126.56	3111.786	3111.814	[ 2]	+0.028	Fe i CH	B
					10	} 13.5	$\{\begin{array}{c} 32100.38\\ 32100.05 \end{array}$	3114.324	3114.316	[ 2]	−0.008	Fe ii CH	B
				10				3114.356	3114.353	[ 1]	−0.003	CH	P
					9	} 13.5	$\{\begin{array}{c} 32073.67\\ 32073.16 \end{array}$	3116.919	3116.917	[ 0]	−0.002	CH	P
				9	8			3116.968	3116.982	[ 1]	+0.014	CH	P
						9	32046.63	3119.548	3119.504	[ 3]	−0.044	Fe i	M
				8	7	9	32045.49*	3119.659	3119.678	[ 1]	+0.019	OH CH Cr i	B
						9	32019.32*	3122.209	3122.219	[ 0]	+0.010	OH CH	B
				7		9	32018.18	3122.320	3122.317	[ 1]	−0.003	Fe i CH	B
					6	11	31991.55	3124.920	3124.918	[ 2]	−0.002	CH OH	B
				6		11	31990.06*	3125.065	3125.053	[ 2]	−0.012	Cr ii—CH	B
					5	9	31963.71	3127.641	3127.671	[ 2d?]	+0.030	|OH CH Ti i?	B
				5		9	31961.62	3127.846	3127.846	[ 1]	0.000	CH	P
					4	7	31935.88	3130.367	3130.415	[ 1]	+0.048	Be ii	M
				4		7	31933.06	3130.543	3130.567	[ 1]	+0.024	|OH Fe ii	M
					3	5	31908.03	3133.099	3133.066	[ 1]	−0.033	‖Fe ii—Sc ii	M
				3		5	31904.23	3133.472	3133.491	[ 0]	+0.019	Zr ii	M
					2	2.5	31880.74	3135.781					A
				2		3	31874.92	3136.354	3136.345	[−1]	−0.009	CH	P
				1		3	31844.93	3139.308	3139.306	[ 0]	−0.002	CH	P
		16	16			} 90	$\{\begin{array}{c} 31802.95\\ 31802.8\\ 31802.75\\ 31802.6\\ 31802.35\\ 31802.2 \end{array}$	3143.452	} 3143.486		$\{\begin{array}{l} +0.034\\ +0.020\\ +0.015\\ -0.001\\ -0.025\\ -0.040 \end{array}$	} V ii CH	M
			15					3143.466					
		15						3143.471					
		17	17					3143.487		[ 2N]			B
			14					3143.511					
		14						3143.526					
			13			} 77	$\{\begin{array}{l} 31801.75\\ 31801.65\\ 31801.45 \end{array}$	3143.570	} 3143.575		$\{\begin{array}{l} +0.005\\ -0.005\\ -0.025 \end{array}$	CH	
		18	18					3143.580		[ 1N]			B
		13						3143.600					
			12			} 61	$\{\begin{array}{l} 31801.05\\ 31800.5\\ 31800.3\\ 31800.25 \end{array}$	3143.640					A
		12						3143.694					A
		19	19					3143.714	} 3143.764		$\{\begin{array}{l} +0.050\\ +0.045\\ -0.019\\ -0.039 \end{array}$	}	M
			11					3143.719					
		11				} 51	$\{\begin{array}{l} 31799.6\\ 31799.4 \end{array}$	3143.783		[ 4]		‖Ti ii CH—OH	B
			10					3143.803					
		10				} 55	$\{\begin{array}{l} 31798.6\\ 31798.5 \end{array}$	3143.88	} 3143.896		$\{\begin{array}{l} +0.02\\ +0.01\\ -0.03 \end{array}$	CH Cr ii	
			9					3143.89		[ 0]			B
		20	20			} 57	$\{\begin{array}{l} 31798.1\\ 31797.6\\ 31797.5 \end{array}$	3143.93					
			8					31,43.98	} 3143.996	[ 2]	$\{\begin{array}{l} +0.02\\ +0.01 \end{array}$	CH Fe i	B
		9						3143.99					
			7			} 55	$\{\begin{array}{l} 31796.7\\ 31796.4\\ 31796.0\\ 31795.9 \end{array}$	3144.07					A
		8						3144.10	}3144.116		$\{\begin{array}{l} +0.02\\ -0.02\\ -0.03 \end{array}$	CH	
			6					3144.14		[ 0]			B
			2					3144.15					A
		7	5			} 74	$\{\begin{array}{l} 31795.3\\ 31795.1\\ 31795.0 \end{array}$	3144.21	} 3144.236		$\{\begin{array}{l} +0.03\\ +0.01\\ 0.00 \end{array}$	CH	
		21	21					3144.23		[ 0]			B
			4, 3					3144.24					
		6				36	31794.1	3144.33	3144.326	[ 0]	0.00	CH OH|	B
		5				25	31793.0	3144.44	3144.453	[ 1]	+0.01	CH Cr i	B
		4				22	31792.0	3144.53	3144.501	[ 1]	−0.03	Fe i CH	B
		3, 22	22			26	31790.93	3144.640	3144.629	[ 0]	−0.011	CH	P
		2				16	31789.78	3144.754	3144.737	[ 1]	−0.017	Ti ii V ii—Fe ii|	M
		1				8	31788.13	3144.917	3144.925	[ 1	+0.008	CH Fe i	B
		23	23			6	31786.06	3145.122	3145.091	[ 3]	−0.031	|Fe i—Cr ii (CH)	M
		24				} 4	$\{\begin{array}{l} 31779.59\\ 31779.12 \end{array}$	$\{\begin{array}{l} 3145.763\\ 3145.809 \end{array}$	3145.791	[ 1]	$\{\begin{array}{l} +0.028\\ -0.018 \end{array}$	Cr ii? CH—CH	B
			24										
		25				} 3	$\{\begin{array}{l} 31771.48\\ 31770.96 \end{array}$	$\{\begin{array}{l} 3146.566\\ 3146.617 \end{array}$	3146.598	[ 1]	$\{\begin{array}{l} +0.032\\ -0.019 \end{array}$	CH—CH OH	B
			25										
		26				2	31761.53	$\{\begin{array}{l} 3147.551\\ 3147.609 \end{array}$	3147.599	[ 1]	$\{\begin{array}{l} +0.048\\ -0.010 \end{array}$	Fe i—CH	B
			26			2	31760.95						
1						4	31759.34	3147.768	3147.784	[ 1]	+0.016	Fe i	M
		27				1	31749.88	3148.706					A
			27			1	31749.32	3148.762	3148.797	[ 0N]	+0.035		M
	2					5	31738.48	3149.837	3149.852	[ 2]	+0.015	OH	M
2						6	31732.62	3150.419	3150.417	[−1]	−0.002	CH	P
	3					6	31709.12	3152.754	3152.737	[ 1Nd?]	−0.017	Co i—CH	B
3						6	31705.16	3153.148	3153.191	[ 3]	+0.043	|Fe i OH	M
	4					7	31680.54	3155.598	3155.622	[ 0]	+0.024	CH	P
4						7	31677.60	3155.891	3155.905	[ 0]	+0.014	CH	P
	5					14	31652.28*	3158.416	3158.403	[ 1]	−0.013	CH	P
5						11	31650.11	3158.632	3158.633	[ 0]	+0.001	CH	P
	6					10	31624.45	3161.195	3161.204	[ 3]	+0.009	Ti ii	M
G						13	31622.73*	3161.367	3161.382	[ 1]	+0.015	Fe i CH	B
	7					12	31596.93	3163.949	3163.930	[ 1]	−0.019	Cr ii—CH	B
7						13	31595.59	3164.083	3164.068	[ 0]	−0.015	CH	P
	8					12	31569.75	3166.673	3166.674	[ 1]	+0.001	CH Fe ii	B
8						13	31568.73	3166.775	3166.767	[ 0]	−0.008	CH	P
	9					14	31542.82	3169.376	3169.366	[ 1]	−0.010	CH	P
9						15	31542.22	3169.438	3169.427	[ 1]	−0.011	CH	P
	10					} 18	$\{\begin{array}{l} 31516.27\\ 31515.92 \end{array}$	3172.047	3172.051	[ 3]	$\{\begin{array}{l} +0.004\\ +0.005 \end{array}$	‖Fe i Cr ii (CH)	M
10								3172.082	3172.087	[ 4]			
11	11					20	31489.89	3174.704	3174.697	[ 2]	−0.007	CH	P
12	12					18	31464.03	3177.313	3177.302	[ 2]	−0.001	Co i—CH	B
13	13					19	31438.33	3179.911	3179.901	[ 0]	−0.010	CH	P
14	14					17	31412.94	3182.481	3182.471	[ 2]	−0.010	CH	P
15	15					15	31387.76	3185.034	3185.022	[ 1N]	−0.012	CH	P
16						}11	$\{\begin{array}{l} 31363.00\\ 31362.71 \end{array}$	$\{\begin{array}{l} 3187.549\\ 3187.578 \end{array}$	3187.556	[ 0Nd?]	$\{\begin{array}{l} +0.007\\ -0.022 \end{array}$	CH—CH	B
	16												
17						} 10	$\{\begin{array}{l} 31338.25\\ 31337.82 \end{array}$	3190.066	3190.042	[ 1]	−0.024	Fe i—CH	B
	17							3190.110	3190.104	[ 1]	−0.006	CH	P
18						} 8	$\{\begin{array}{l} 31313.65\\ 31313.07 \end{array}$	3192.573	3192.534	[−1]	−0.039	—CH?	B
	18							3192.632	3192.617	[−1]	−0.015	CH	P
19						6	31289.07	3195.080	3195.085	[ 0]	+0.005	CH OH	B
	19					7	31288.28	3195.161	3195.140	[−1]	−0.021	Ru ii CH	B
20						4	31264.33	3197.609	3197.596	[−1]	−0.013	Y ii?—CH	B
	20					8	31263.44*	3197.700	3197.710	[ 1N]	+0.010	CH	P
21						3	31239.60	3200.140	3200.137	[−1]	−0.003	CH	P
	21					4	31238.63	3200.240	3200.295	[ 2Nd?]	+0.055	Y ii—	M
22						3	31214.62	3202.701	3202.695	[ 0]	−0.006	CH OH	B
	22					4	31213.57	3202.809	3202.822	[ 0]	−0.013	CH	P
23						1.5	31189.12	3205.320	3205.333	[−1]	+0.013	CH	P
	23					2	31188.04	3205.431	3205.408	[ 2]	−0.023	Fe i—CH	B
24						1	31163.39	3207.967	3207.989	[ 0]	+0.022	CH—	B
	24					1.5	31162.11	3208.098	3208.094	[ 0]	−0.004	CH	P
25						1	31136.68	3210.719	3210.724	[−1]	+0.005	CH OH	B
	25					1	31135.45	3210.845	3210.836	[ 2]	−0.009	Fe i	M
26						1	31108.98	3213.577	3213.564	[−2]	−0.013	Ti ii Fe i	M
	26					1	31107.64	3213.716	3213.694	[−1]	−0.022		M
27						1	31080.42	3216.530	3216.546	[ 0]	+0.016	Cr ii	M
	27					1	31079.03	3216.674	3216.694	[ 1]	+0.020	Y ii	M
28						0.5	31050.36	3219.645					A
	28					1	31049.00	3219.786	3219.805	[ 3]	+0.019	Fe i	M

* Blend.

**Table 11:** CH in the Solar Spectrum C ^2^Σ^+^—X ^2^Π (1, 1)

Laboratory						Sun				

P	Q	R	Intensity	Wave number cm^−1^	Wavelength A	Wavelength A	Disk int. Rowl. est.	⊙—lab. A	Solar identification	Notes
		19	1	32050.51	3119.171	3119.198	[ 1Nd?]	+0.027		M
		18	(CH)	32045.67*	3119.642	3119.678	[ 1]	+0.036	OH CH Cr i	M
		17	1	32038.34	3120.355	3120.372	[ 3 ]	+0.017	Cr ii	M
		16	1.5	32029.60	3121.207	3121.160	[ 4 ]	−0.047	V ii	M
		15	(CH)	32019.19*	3122.222	3122.219	[ 0 ]	−0.003	OH CH	M
		14	2	32006.57	3123.453	3123.443	[−1 ]	−0.010	OH	M
		13	(CH)	31990.65*	3125.007	3124.998	[ 4 ]	−0.009	Cr ii	M
		12	4.5	31974.16	3126.619	3126.617	[ 1 ]	−0.002	CH OH—OH	B
		11	3	31956.69	3128.328	3128.289	[ 1 ]	−0.039	Sc ii OH |	M
		10	(OH)	31936.86	3130.271	3130.267	[ 3 ]	−0.004	|V ii—OH	M
		8	(OH)	31895.65	3134.315	3134.337	[ 1 ]	+0.022	|OH Cr ii	M
		7	1.5	31873.48	3136.495	3136.506	[ 2 ]	+0.011	V ii	M
		6	1.3	31850.58	3138.751	3138.786	[ 0 ]	+0.035		A
		5	1.5	31826.96	3141.080	3141.106	[−1 ]	+0.026	CH?	P
	2		2	31670.8	3156.57	3156.565	[ 0 ]	−0.01	—CH	B
	3			31670.2	3156.63					A
	4		2	31669.0	3156.75	3156.727	[ −1 ]	−0.02	CH	P
	5		2	31667.2	3156.93	3156.916	[ 0 ]	−0.01	—CH	B
	6		6	31665.2	3157.13	3157.143	[ 1 ]	+0.01	|OH—Fe ip	M
	7		3	31663.0	3157.35					A
	8		4	31660.1	3157.64	3157.634	[−1 ]	−0.01	CH	P
	9		4	31658.8	3157.77	3157.751	[ 0 ]	−0.02	CH OH?	B
	10		(CH)	31652.9*	3158.36	3158.351	[ 0 ]	−0.01	Fe iip—	M
	11		6	31648.4	3158.80	3158.783	[ 1 ]	−0.02	Co i CH	B
	12		6	31643.3	3159.31	3159.349	[ 0 ]	+0.04	Fe iip?—V ii?	M
	13		6	31637.2	3159.92	3159.935	[ 0N ]	+0.02	CH—	B
	14		5	31630.3	3160.61	3160.612	−1	0.00	Cr i CH	B
	15		(CH)	31622.2*	3161.42	3161.423	[ 0 ]	0.00		M
	16		6	31612.9	3162.35	3162.353	[ 1 ]	0.00	Fe i CH	B
	17		4	31602.1	3163.43	3163.423	[ 2 ]	−0.01	Nb ii—CH	B
	18		4	31589.5	3164.69	3164.685	[ 1 ]	−0.01	Mn ii—CH	B
	19		3	31575.1	3166.14	3166.130	[ 0 ]	−0.01	CH	P
	20		3	31558.4	3167.81	3167.790	[ 1 ]	−0.02	Fe i—CH	B
	21		(CH)	31540.6	3169.60	3169.616	[ 0 ]	+0.02	OH	M
6			2	31503.07	3173.376	3173.408	[ 1 ]	+0.032	Fe i	M
7			2	31473.45	3176.362	3176.351	[ 1 ]	−0.011	Fe i	M
8			2	31444.83	3179.253					A
9			3	31417.00	3182.070	3182.061	[ 1 ]	−0.009	Fe i—CH	B
10			(CH)	31385.15	3185.299	3185.327	[ 2 ]	+0.028	Fe ii	M
11			4	31355.47	3188.314	3188.335	[ 0 ]	+0.021	CH	P
12			4	31325.15	3191.400	3191.414	[ 0 ]	+0.014	Fe ip CH	B
13			4	31294.43	3194.533	3194.524	[−1 ]	−0.009	CH	P
14			(CH)	31263.55*	3197.689	3197.710	[ 1N ]	+0.021	CH	M
15			3	31231.66	3200.954	3200.962	[ 1 ]	+0.008	OH	M
16			2.5	31199.10	3204.295	3204.284	[ 2Nd?]	−0.011		M
17			2	31165.79	3207.720	3207.711	[ 0 ]	−0.009	Fe i	M
18			1.5	31131.80	3211.222	3211.209	[−1 ]	−0.013	CH?	P
19			1	31096.37	3214.881	3214.864	[−2 ]	−0.017	CH?	P
20				31059.68	3218.678	3218.684	[−1 ]	+0.006	Cr i	M
21				31021.21	3222.670					A

* Blend.

## Footnotes

1 Figures in brackets indicate the literature references at the end of this paper.
